# Cancer risk among insulin users: comparing analogues with human insulin in the CARING five-country cohort study

**DOI:** 10.1007/s00125-017-4312-5

**Published:** 2017-06-01

**Authors:** Anna But, Marie L. De Bruin, Marloes T. Bazelier, Vidar Hjellvik, Morten Andersen, Anssi Auvinen, Jakob Starup-Linde, Marjanka K. Schmidt, Kari Furu, Frank de Vries, Øystein Karlstad, Nils Ekström, Jari Haukka

**Affiliations:** 1 Department of Public Health Clinicum, University of Helsinki, Tukholmankatu 8B, P.O. Box 20, 00014 Helsinki, Finland; 2 Division of Pharmacoepidemiology and Clinical Pharmacology, Utrecht Institute for Pharmaceutical Sciences, Utrecht University, Universiteitsweg 99, 3584 CG Utrecht, the Netherlands; 3 Copenhagen Centre for Regulatory Science (CORS), Department of Pharmacy, University of Copenhagen, Copenhagen, Denmark; 4 Department of Pharmacoepidemiology, Norwegian Institute of Public Health, Oslo, Norway; 5 Centre for Pharmacoepidemiology, Karolinska Institutet, Stockholm, Sweden; 6 Research Unit of General Practice, University of Southern Denmark, Odense, Denmark; 7 Department of Drug Design and Pharmacology, University of Copenhagen, Copenhagen, Denmark; 8 Department of Epidemiology, School of Health Sciences, University of Tampere, Tampere, Finland; 9 Department of Endocrinology and Internal Medicine, Aarhus University Hospital THG, Aarhus, Denmark; 10 Division of Molecular Pathology, The Netherlands Cancer Institute – Antoni van Leeuwenhoek Hospital, Amsterdam, the Netherlands; 11 Division of Psychosocial Research and Epidemiology, The Netherlands Cancer Institute – Antoni van Leeuwenhoek Hospital, Amsterdam, the Netherlands; 12 The Netherlands Department of Clinical Pharmacy and Toxicology, Maastricht University Medical Centre, Maastricht, the Netherlands; 13 The Netherlands Research Institute CAPHRI, Maastricht University, Maastricht, the Netherlands; 14 The Netherlands MRC Lifecourse Epidemiology Unit, University of Southampton, Southampton, UK

**Keywords:** Cancer risk, Cohort study, Common data model, Cumulative treatment time, Detemir, Glargine, Human insulin, Insulin analogues, New insulin user, Poisson model, Rate ratio, Semi-aggregate, Site-specific

## Abstract

**Aims/hypothesis:**

The aim of this work was to investigate the relationship between use of certain insulins and risk for cancer, when addressing the limitations and biases involved in previous studies.

**Methods:**

National Health Registries from Denmark (1996–2010), Finland (1996–2011), Norway (2005–2010) and Sweden (2007–2012) and the UK Clinical Practice Research Datalink database (1987–2013) were used to conduct a cohort study on new insulin users (*N* = 327,112). By using a common data model and semi-aggregate approach, we pooled individual-level records from five cohorts and applied Poisson regression models. For each of ten cancer sites studied, we estimated the rate ratios (RRs) by duration (≤0.5, 0.5–1, 1–2, 2–3, 3–4, 4–5, 5–6 and >6 years) of cumulative exposure to insulin glargine or insulin detemir relative to that of human insulin.

**Results:**

A total of 21,390 cancer cases occurred during a mean follow-up of 4.6 years. No trend with cumulative treatment time for insulin glargine relative to human insulin was observed in risk for any of the ten studied cancer types. Of the 136 associations tested in the main analysis, only a few increased and decreased risks were found: among women, a higher risk was observed for colorectal (RR 1.54, 95% CI 1.06, 2.25) and endometrial cancer (RR 1.78, 95% CI 1.07, 2.94) for ≤0.5 years of treatment and for malignant melanoma for 2–3 years (RR 1.92, 95% CI 1.02, 3.61) and 4–5 years (RR 3.55, 95% CI 1.68, 7.47]); among men, a lower risk was observed for pancreatic cancer for 2–3 years (RR 0.34, 95% CI 0.17, 0.66) and for liver cancer for 3–4 years (RR 0.36, 95% CI 0.14, 0.94) and >6 years (RR 0.22, 95% CI 0.05, 0.92). Comparisons of insulin detemir with human insulin also showed no consistent differences.

**Conclusions**/**interpretation:**

The present multi-country study found no evidence of consistent differences in risk for ten cancers for insulin glargine or insulin detemir use compared with human insulin, at follow-up exceeding 5 years.

**Electronic supplementary material:**

The online version of this article (doi:10.1007/s00125-017-4312-5) contains peer-reviewed but unedited supplementary material, which is available to authorised users.

## Introduction

Diabetes mellitus and cancer are common diseases with rising incidence and prevalence globally [1, 2]. Diabetes is associated with an increased risk for certain cancers [3] and the pattern and magnitude of the excess risk are generally similar for type 1 and 2 diabetes [4, 5]. It has been suggested that certain diabetes risk factors, as well as glucose-lowering medications, may contribute to this association [6]. In 2009, the publication of four observational studies [7–10] sparked concerns about insulin glargine as a potential modifier of cancer risk [11].

The oncogenic potential of various insulin analogues has been suggested by preclinical safety evaluations showing that IGF and insulin receptor signalling pathways, which are essential for mitogenic potency, are affected by ligand-specific receptor dynamics, depending on the cell type [12]. Initial observational studies [7–10] were criticised for limitations and biases [11, 13, 14] such as short follow-up, inclusion of prevalent insulin users and time-lag bias [15]. Further attempts to rule out or confirm the association yielded inconsistent findings, emphasising the importance of properly designed and conducted observational studies [16].

The evidence from the observational studies remains inconsistent [17, 18], particularly due to the involvement of methodological drawbacks, such as time-related biases and selection bias [15]. Moreover, the findings from studies that ignore dose–effect aspects, time-varying character of exposure, site-specific risks or long latency of cancer are limited in their biological plausibility [16, 19]. It was thought that RCTs, the gold standard of treatment comparisons, would disentangle the association. However, the ORIGIN trial, which showed a null effect [20] and constitutes perhaps the strongest RCT evidence concerning the use of insulin analogues and cancer outcomes, has been criticised for evaluation of cancer risk with respect to the initial insulin glargine allocation but not the cumulative insulin glargine use [21]. Overall, there is little robust evidence from observational studies or RCTs to draw firm conclusions.

The present observational study, which is a part of the Cancer Risk and Insulin Analogues (CARING) project, sought to investigate the effect of exposure to insulin glargine or insulin detemir on cancer risk as compared with that of human insulin, mitigating through study design and analytical approaches the limitations and biases involved in the previous studies.

## Methods

### Overview

This cohort study on new insulin users was conducted using nationwide data from the Norwegian, Swedish, Danish and Finnish National Health Registries, as well as data from UK general practitioners in the Clinical Practice Research Datalink (CPRD). Project partners obtained ethics approval from their respective authorities. The study protocol, where data sources and study cohorts are described in more detail, was registered in the European Network of Centres for Pharmacoepidemiology and Pharmacovigilance (ENCePP) electronic register of studies [22]. To synchronise definitions of demographics, exposures, outcomes and confounders, a common data model and concept dictionary were developed. Table 1 shows the study design and methodological approaches to the data analysis that we implemented to mitigate different types of selection, information and time-related biases often inherent in the observational research [15, 23–25].

**Table 1:** Methodological shortcomings and biases mitigated, addressed by checking the robustness of the results or disentangled by the design and analytical features used in the study

Design/analytical feature	Selection bias		Information bias			Time-related bias		Confounding bias	
	Healthcare access bias^a^	Prevalent user bias^b^	Misclassification of exposure^c^	Misclassification of outcome^c^, detection bias^d^	Protopathic bias (reverse causation)^e^	Immortal time bias^f^	Time-lag bias^g^, time-window bias^h^	Confounding by indication^i^	Residual confounding^j^
Adjustment for time since start of insulin use	–	–	Mitigated	–	–	–	Mitigated^g^	–	Mitigated
Active-comparator approach	–	–	–	Mitigated^d^	–	Mitigated	Mitigated^g^	Mitigated	Mitigated
Cumulative exposure definition	–	–	Mitigated	–	Disentangled	–	–	Mitigated	–
Nationwide Nordic drug registers	Mitigated	–	–	–	–	–	–	–	–
Nationwide Nordic cancer registers	–	–	–	Mitigated^c^	–	–	–	–	–
New-user design	–	Mitigated	Mitigated	–	–	Mitigated	Mitigated^h^	Mitigated	–
Sensitivity analysis	Checked^k^	–	–	Checked^c,k^	–	–		–	Checked^l^
Time-varying exposure definition	–	–	Mitigated	–	Disentangled	Mitigated	Mitigated^h,m^	Mitigated	

^a^Healthcare access bias: differential degree of access to the healthcare among patients

^b^Prevalent user bias: risk of outcome is considerably higher or lower during the early period of drug therapy; inclusion of prevalent users may distort the association between the use of drug and outcome (including prevalent users may also introduce confounding)

^c^Misclassification (measurement) bias: inaccurate measurement or classification of key study variables, such as exposure, outcome or confounders: misclassification of exposure may arise from use of too simple (binary) exposure definition, especially for the complex pattern of use; misclassification of outcome may occur due to the use of incomplete records to identify events

^d^Detection bias: different probability of outcome detection during the follow-up in the compared groups

^e^Protopathic bias: symptoms treated by a drug are the manifestation of the yet-undiagnosed disease of interest

^f^Immortal time bias arises because of exclusion or misclassification of the follow-up time between the cohort entry and first exposure to a drug, before which the outcome of interest cannot occur

^g^Time-lag bias: compared treatments are commonly used at different stages of the disease (first-line therapy vs second- or third-line therapy)

^h^Time-window bias: unequal opportunity to become exposed between the compared groups owing to the time-window differential

^i^Confounding by indication: differences between compared treatments with respect to their indications (or contraindications)

^j^Residual confounding: the distortion that remains after controlling for confounding due to the unmeasured/uncontrolled confounders

^k^Restriction to the Nordic cohorts

^l^Restriction to the individuals with type 2 diabetes

^m^Restriction to the calendar period from 2000 onwards

### Data sources, selection and follow-up of participants

National health registries in the Nordic countries comprise computerised records for the entire population of 26 million people, each of whom are assigned a unique personal identification number. The cancer registries have a long tradition of providing comparable and high-quality data with almost 100% coverage of incident cancer cases [26]. Prescription registries, established in 1995 in Denmark and Finland, in 2004 in Norway and in 2005 in Sweden, have provided ample data for pharmaco-epidemiological research [27]. The CPRD, a large computerised database established in the UK in 1987, contains anonymised medical records, including demographics, prescriptions and cancer diagnoses, that are considered to be of good quality [28]. Currently, 4.4 million individuals, 6.9% of the UK population, meet the quality criteria and are broadly representative of the entire population with regard to demographic characteristics [29].

Within the study period (Table 2) defined as the period of valid data collection [22], we identified all individuals having at least one insulin purchase (Nordic countries) or prescription (CPRD). Nordic cohorts were linked with the registered data on cancer, death and emigration; data for the British cohort were compiled from information on cancer and death available from the CPRD. New insulin users, who were defined based on a 1 year lead-in period, were included if they had no history of cancer (except non-melanoma skin cancer) and were aged ≥18 years on the first prescription for any insulin (index date). Follow-up started at index date and ended at the date of emigration (Sweden, Denmark and Norway) or transfer out of the CPRD, diagnosis of any cancer (excluding non-melanoma skin cancer), death or end of follow-up, whichever occurred first.

**Table 2:** Baseline and follow-up characteristics of the study cohorts of new users of insulin

Characteristic	Denmark (*N* = 66,698)	Finland (*N* = 105,945)	Norway (*N* = 21,541)	Sweden (*N* = 85,319)	UK (CPRD) (*N* = 47,609)
Study period^a^	1996–2010	1996–2011	2005–2010	2007–2012	1987–2013
Male sex, *n* (%)	38,292 (57)	57,691 (55)	12,053 (56)	48,931 (57)	25,589 (54)
Age years, mean (SD)^b^	60.1 (16.0)	61.6 (15.9)	57.3 (17.8)	64.0 (16.4)	59.3 (16.3)
Age years, *n* (%)^b^					
18–30	3193 (4.8)	4481 (4.2)	1634 (7.6)	3121 (3.6)	2555 (5.4)
30–40	5075 (7.6)	7317 (6.9)	2653 (12.3)	4924 (5.8)	4540 (9.5)
40–50	8334 (12.5)	11,095 (10.5)	3088 (14.3)	8383 (9.8)	5825 (12.2)
50–60	14,432 (21.6)	22,928 (21.6)	4248 (19.7)	14,870 (17.4)	9351 (19.6)
60–70	16,306 (24.5)	25,254 (23.8)	4220 (19.6)	21,378 (25.1)	11,661 (24.5)
70–80	12,527 (18.8)	22,110 (20.9)	3194 (14.8)	17,176 (20.1)	9505 (20.0)
80+	6831 (10.2)	12,760 (12.0)	2504 (11.6)	15,466 (18.1)	4172 (8.8)
Follow-up time, years					
Mean (SD)	5.3 (3.9)	5.6 (3.9)	2.7 (1.8)	2.7 (1.8)	5.7 (4.3)
Median (interquartile range)	4.5 (1.9, 7.8)	4.7 (2.3, 8.3)	2.5 (1.1, 4.1)	2.6 (1.1, 4.1)	4.7 (1.9, 8.4)
No. of person-years/1000, all (male sex)	331.2 (184.4)	589.1 (316.9)	57.8 (32.4)	226.6 (131.3)	265.3 (141.6)
Ever-use, *n* (%)^c^					
Human insulin	54,216 (81)	68,894 (65)	17,579 (82)	48,976 (57)	23,183 (49)
Insulin glargine	7151 (11)	43,741 (41)	1447 (7)	15,138 (18)	15,374 (32)
Insulin detemir	9520 (14)	24,593 (23)	868 (4)	4367 (5)	7373 (15)
Other insulin	33,388 (50)	48,280 (46)	14,376 (67)	53,810 (63)	27,491 (58)
Baseline use, *n* (%)					
HRT^d^	5187 (18)	6546 (14)	1641 (17)	6621 (18)	1530 (7)
NSAID^e^	17,800 (27)	29,609 (28)	5437 (25)	16,485 (19)	8935 (18)
Any oral glucose-lowering therapy	49,569 (74)	83,935 (79)	15,051 (70)	62,522 (73)	37,239 (78)
Statin	22,948 (34)	38,493 (36)	9309 (43)	39,635 (46)	24,188 (51)

^a^Start of study period defined according to the start of prescription registry (Nordic countries) or start of valid data collection (CPRD)

^b^Age at baseline

^c^Ever-use of specific insulin during the follow-up

^d^Female sex only

^e^Based on prescriptions only

### Cancer outcomes, insulin treatments and potential confounders

We relied on coding dictionaries, compiled according to different coding systems (ICD-7, ICD-9 [www.icd9data.com/2007/Volume1], ICD-10 [www.who.int/classifications/icd/en/] and ICD-O-3 in the Nordic countries; Read code system in the CPRD), to identify incident cancer cases defined as the first occurrence of any cancer type [22]. Multiple cancers diagnosed on the same date were considered as distinct site-specific endpoints. Our primary interest was in ten site-specific cancers. Based on NORDCAN data (cancer statistics from Nordic countries) [26], we selected the eight cancer types with the highest incidence rates (ICD-10 codes): trachea and lung (C33, C34), melanoma of skin (C43), bladder (C67), colorectal (C18-21), non-Hodgkin lymphoma (C82-86, C88.4), breast (C50), endometrial (C54) and prostate (C61). Liver (C22) and pancreatic (C25) cancers were also included because of their strong association with diabetes. As a secondary outcome of interest, we investigated the first occurrence of any cancer.

Based on Anatomical Therapeutic Chemical (ATC) classification codes [30] (British National Formulation codes for the CPRD), we identified users of human insulin (A10AC01, A10AB01, A10AD01, A10AE01, A10AF01) and the insulin analogues insulin glargine (A10AE04) and insulin detemir (A10AE05). Any other insulins and analogues were considered as a single group. Prescription data form the Nordic registries included the date and amount purchased, in defined daily doses (DDDs) [30], but no information on individual dosage. For the CPRD cohort, we derived DDDs from the dosage information (substance strength and amount) contained within prescription data. We assumed a daily consumption of 1 DDD per day and transformed each drug record into a period covered by the number of DDDs.

For each insulin type of interest, we defined insulin exposure time-dependently as a cumulative treatment time. After splitting the individual follow-up period into intervals of 120 days, the exposure at the beginning of each interval was updated. The exposure began on the date of first prescription/purchase, after which point an individual was considered exposed. Cumulative treatment time accrued until exposure stopped and remained unchanged, unless treatment was resumed (see electronic supplementary material [ESM] Methods). We then divided cumulative treatment time into half-year categories for the first year and 1 year categories for longer exposure; the last categories were >6 years for the broadly categorised exposure and 9–10 years for the finely categorised exposure. In addition, each exposure variable incorporated a non-exposed category assigned to individuals remaining unexposed to the specific insulin.

We considered only confounders available in all five datasets [22]. In addition to age, sex and calendar time, this included use of non-insulin glucose-lowering drugs (ATC code A10B), statins (C10A), nonsteroidal anti-inflammatory drugs (NSAIDs; M01A) and hormone replacement treatment (HRT; G03), defined as at least one prescription within 1 year before the index date. We also derived several other potential confounders: type 1 diabetes mellitus was assigned to those aged ≤30 years with no non-insulin glucose-lowering drug on the index date; type 2 diabetes mellitus was assigned to those aged ≥40 years with or without non-insulin glucose-lowering drugs; unspecified diabetes type was assigned to the rest of the cohort. We specified the duration of insulin-treated diabetes as time since the index date (in 1 year intervals) and defined menopausal status time-dependently based on cut-off of 50 years of age. Furthermore, the country of the data origin served as a covariate.

### Statistical methods

The individual-level data from the five cohorts were standardised by each research partner locally using the common data model. We then conducted centralised analyses by uploading the unified data to a server at Statistics Denmark, where for each cohort we constructed the individual-level dataset to assess insulin exposure and other variables in exactly the same way. We employed a semi-aggregate level approach [31] to combine the datasets, which were tabulated by cancer site as the number of cancer cases and person-years aggregated by categorical variables. To estimate the incidence rates, we fitted multivariable Poisson regression models to the event numbers with the natural log of person-years as an offset. Each model included all three time-dependent insulin exposure variables and was adjusted for time-dependent age and duration of insulin-treated diabetes, sex (not in the sex-stratified analysis), baseline calendar time, use of non-insulin antidiabetic drugs (NIADs), other co-medication (when relevant [22]) and country.

We conducted an active-comparator analysis [25], where the drug of interest is compared with another drug commonly used for the same indication rather than with no treatment. Inclusion of all three insulin exposures in the same model allowed us to calculate the rate ratios (RRs) and 95% CIs for a particular exposure category by contrasting the incidence rates, which were estimated for each insulin type and duration. In the primary analyses, we examined sex- and site-specific cancer endpoints without separating between diabetes types and using insulin exposures with a broader category (>6 years) for the longer cumulative treatment time. For the secondary analyses, we performed similar evaluations without stratifying on sex and using insulin exposures with finer categories.

### Sensitivity analysis

We also performed several sensitivity analyses. We restricted the analyses to those who met type 2 diabetes criteria to check whether the results change by diabetes type (data for individuals with type 1 diabetes were limited). In Europe, marketing authorisation for the long-acting insulin analogues insulin glargine and insulin detemir was granted in June 2000 and June 2004, respectively [32, 33]. Coincidentally, usage of two already-approved rapid-acting insulin analogues, insulin lispro and insulin aspart, gained popularity in the early 2000s. To account for the changes in the profiles of new insulin users, we excluded those entered before 2000. By excluding the CPRD, we addressed the potential of underestimating cancer incidence due to case ascertainment through the CPRD only without linkage to the national cancer registration data. For breast and endometrial cancer, we further adjusted for menopausal status.

We used version 3.2.2 of R (www.R-project.org) [34] to perform all statistical analyses, the Epi package, version 1.1.71 (https://cran.r-project.org/web/packages/Epi/index.html) [35] to carry out exposure calculations and the forestplot package, version 1.7 (https://cran.r-project.org/web/packages/forestplot/index.html) [36] for the graphical output.

## Results

In the five cohorts totalling 327,112 new insulin users, men predominated and the mean age at baseline varied between 57 and 64 years (Table 2). For the combined data, the mean follow-up time was 4.6 years (median 3.7, interquartile range 1.7–6.3). At the end of follow-up, there was 212,848, 82,851 and 46,721 ever-users of human insulin, insulin glargine and insulin detemir, respectively. In all cohorts, human insulin predominated in ever-use patterns. Ever-use of insulin glargine and insulin detemir was most common in the Finnish cohort, as was the baseline use of non-insulin glucose-lowering therapy. Baseline use of other medication also differed between the cohorts.

A total of 1.47 million person-years accumulated and 21,390 new cancer cases occurred during the follow-up. Table 3 shows country- and sex-specific crude incidence rates for the ten site-specific cancers and any cancer. Prostate cancer in men and breast cancer in women showed the highest incidence rates in all cohorts except the Norwegian, where pancreatic cancer was the most common cancer. About 32% of all cancer cases and the majority of pancreatic cancer cases (63%) were diagnosed during the first year of insulin treatment.

**Table 3:** Sex- and site-specific numbers of cancer cases, crude incidence rates with 95% confidence intervals

Cancer type	Denmark		Finland		Norway		Sweden		UK (CPRD)	
	No.	IR (95% CI)	No.	IR (95% CI)	No.	IR (95% CI)	No.	IR (95% CI)	No.	IR (95% CI)
Liver cancer										
Men	144	0.78 (0.66, 0.92)	308	0.97 (0.87, 1.09)	15	0.46 (0.26, 0.76)	89	0.68 (0.54, 0.83)	74	0.52 (0.41, 0.65)
Women	28	0.19 (0.13, 0.28)	102	0.37 (0.31, 0.45)	<6	NS	23	0.24 (0.15, 0.36)	21	0.17 (0.11, 0.26)
Pancreatic cancer										
Men	315	1.71 (1.52, 1.91)	531	1.68 (1.54, 1.82)	76	2.35 (1.85, 2.94)	238	1.81 (1.59, 2.06)	129	0.91 (0.76, 1.08)
Women	233	1.59 (1.39, 1.80)	417	1.53 (1.39, 1.69)	64	2.52 (1.94, 3.21)	199	2.09 (1.81, 2.40)	109	0.88 (0.72, 1.06)
Lung cancer										
Men	466	2.53 (2.30, 2.77)	623	1.97 (1.81, 2.13)	55	1.70 (1.28, 2.21)	192	1.46 (1.26, 1.68)	218	1.54 (1.34, 1.76)
Women	244	1.66 (1.46, 1.88)	174	0.64 (0.55, 0.74)	33	1.30 (0.89, 1.82)	112	1.17 (0.97, 1.41)	116	0.94 (0.77, 1.12)
Melanoma of skin										
Men	59	0.32 (0.24, 0.41)	130	0.41 (0.34, 0.49)	27	0.83 (0.55, 1.21)	80	0.61 (0.48, 0.76)	69	0.49 (0.38, 0.62)
Women	53	0.36 (0.27, 0.47)	77	0.28 (0.22, 0.35)	9	0.35 (0.16, 0.67)	45	0.47 (0.34, 0.63)	35	0.28 (0.20, 0.39)
Bladder cancer										
Men	106	0.57 (0.47, 0.70)	281	0.89 (0.79, 1.00)	43	1.33 (0.96, 1.79)	183	1.39 (1.20, 1.61)	117	0.83 (0.68, 0.99)
Women	34	0.23 0.16, 0.32)	77	0.28 (0.22, 0.35)	<6	NS	40	0.42 (0.30, 0.57)	28	0.23 (0.15, 0.33)
Colorectal cancer										
Men	423	2.29 (2.08, 2.52)	484	1.53 (1.39, 1.67)	68	2.10 (1.63, 2.66)	312	2.38 (2.12, 2.66)	219	1.55 (1.35, 1.77)
Women	258	1.76 (1.55, 1.99)	366	1.34 (1.21, 1.49)	46	1.81 (1.32, 2.41)	145	1.56 (1.31, 1.83)	102	0.82 (0.67, 1.00)
Non-Hodgkin lymphoma										
Men	52	0.28 (0.21, 0.37)	180	0.57 (0.49, 0.66)	14	0.43 (0.24, 0.73)	68	0.52 (0.40, 0.66)	31	0.22 (0.15, 0.31)
Women	47	0.32 (0.24, 0.43)	132	0.48 (0.41, 0.58)	7	0.28 (0.11, 0.57)	46	0.48 (0.35, 0.64)	35	0.28 (0.20, 0.39)
Breast cancer^a^	462	3.15 (2.87, 3.45)	729	2.68 (2.49, 2.88)	51	2.00 (1.49, 2.64)	250	2.62 (2.31, 2.97)	301	2.43 (2.17, 2.72)
Endometrial cancer	152	1.04 (0.88, 1.21)	289	1.06 (0.94, 1.19)	29	1.14 (0.76, 1.64)	107	1.12 (0.92, 1.36)	57	0.46 (0.35, 0.60)
Prostate cancer	501	2.72 (2.48, 2.97)	1339	4.23 (4.00, 4.46)	100	3.09 (2.51, 3.76)	582	4.43 (4.08, 4.81)	290	2.05 (1.82, 2.30)
Any cancer										
Men	2733	14.82 (14.27, 15.39)	5315	16.77 (16.33, 17.23)	542	16.74 (15.36, 18.21)	2272	17.31 (16.60, 18.04)	1918	13.54 (12.94, 14.16)
Women	1950	13.29 (12.70, 13.89)	3618	13.29 (12.86, 13.73)	333	13.09 (11.73, 14.58)	1361	14.27 (13.52, 15.05)	1348	10.89 (10.32, 11.49)

^a^Women only

IR, incidence rate; NS, not shown (in cells where number of events <6 the IR and CI are not shown according to confidentiality principles)

**Fig. 1:**
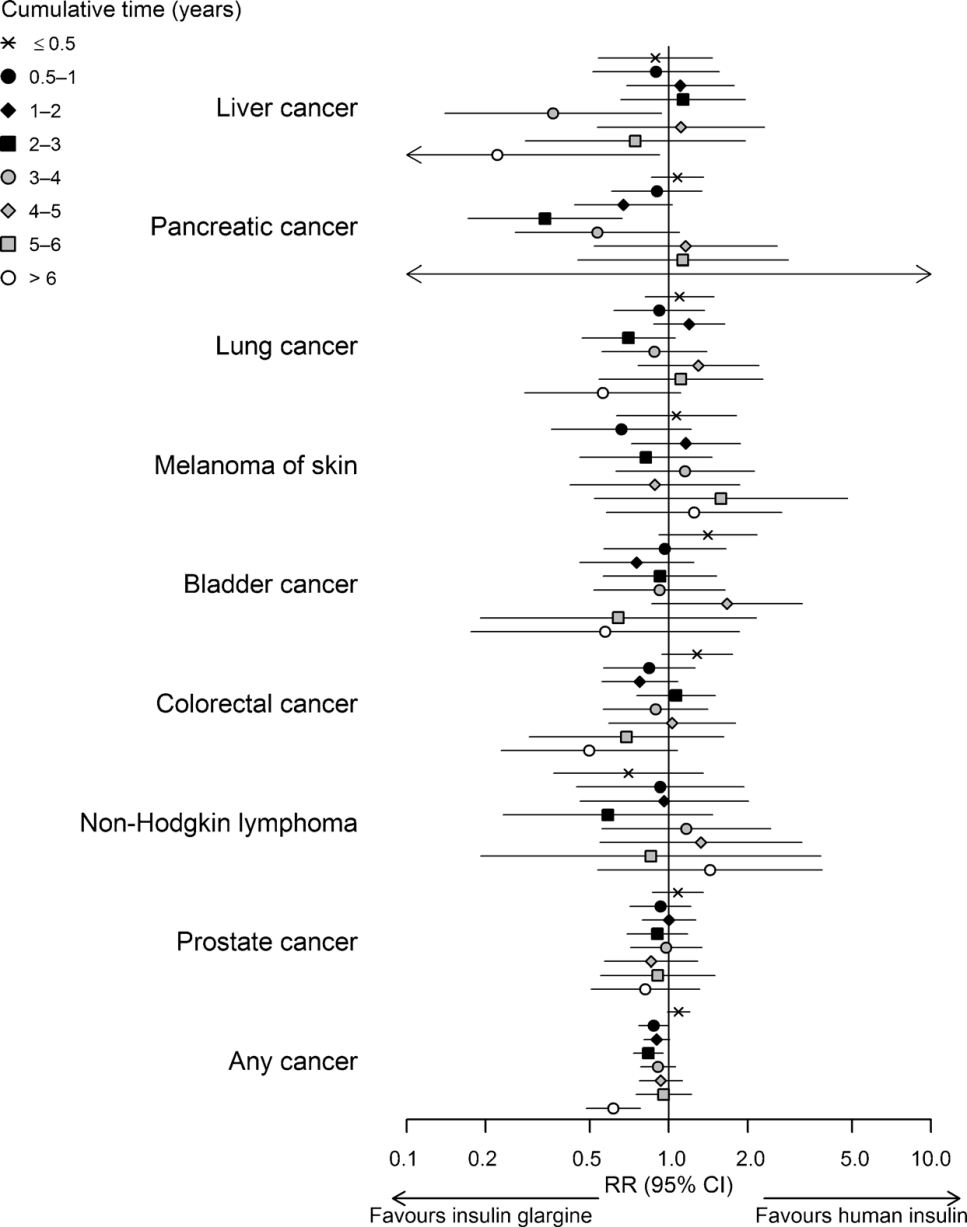
RR (adjusted for age, calendar time, NIADs, duration of insulin-treated diabetes, country; for liver and colorectal cancers, additional adjustment for relevant co-medications) with 95% CI for site-specific cancers and any cancer in men by cumulative treatment time (years) on insulin glargine vs human insulin

**Fig. 2:**
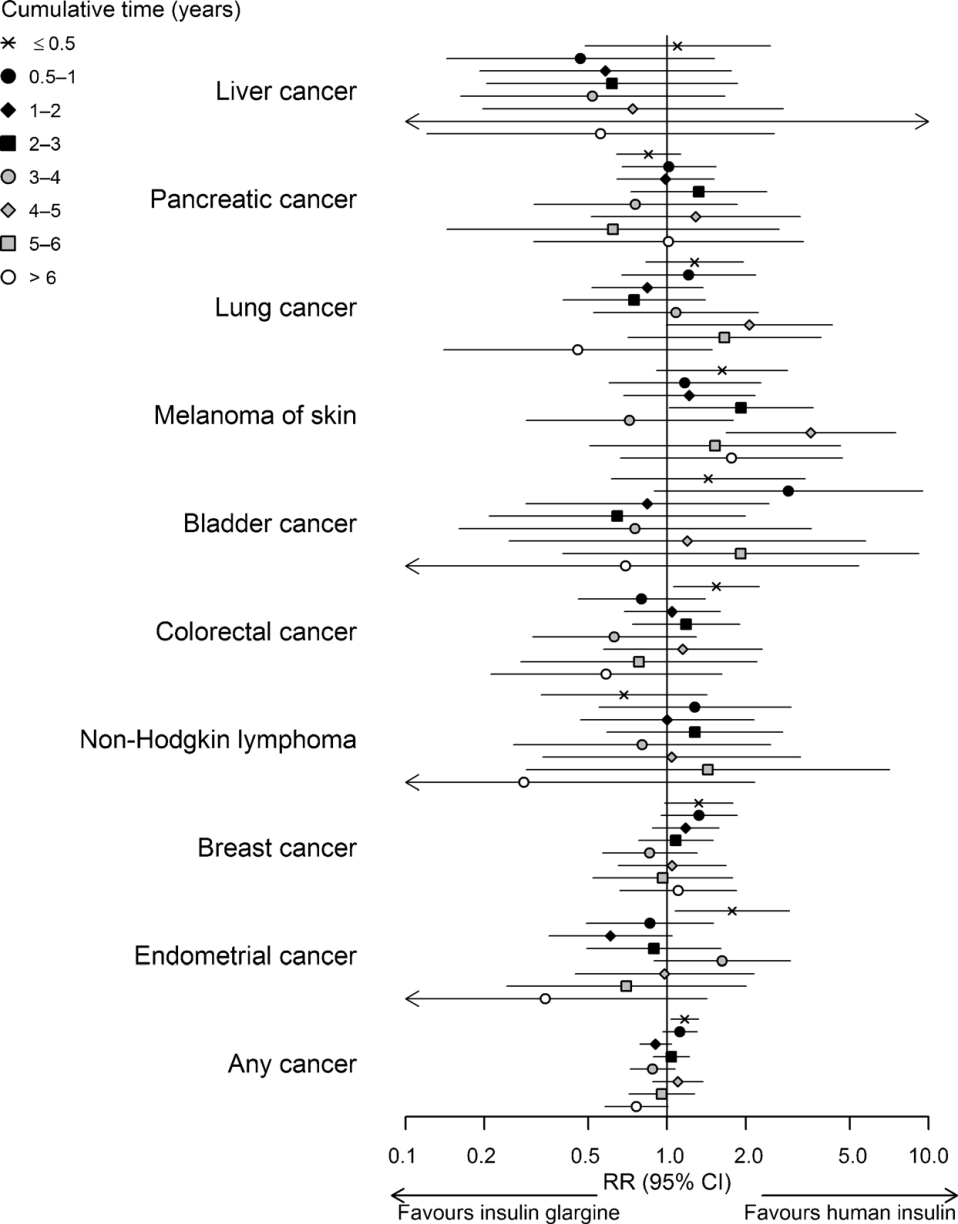
RR (adjusted for age, calendar time, NIADs, duration of insulin-treated diabetes, country; for liver, colorectal, breast and endometrial cancers, additional adjustment for relevant co-medications) with 95% CI for site-specific cancers and any cancer in women by cumulative treatment time (years) on insulin glargine vs human insulin

The results of the sex- and site-specific analyses showed no systematic differences across sites and exposure categories (Figs 1, 2 and ESM Table 1). In women, a higher risk for colorectal (RR 1.54, 95% CI 1.06, 2.25) and endometrial (RR 1.78, 95% CI 1.07, 2.94) cancer was observed for the first half-year of cumulative treatment time on insulin glargine relative to that on human insulin and a higher risk for melanoma of skin for 2–3 years (RR 1.92, 95% CI 1.02, 3.61) and 4–5 years (RR 3.55, 95% CI 1.68, 7.47). In men, similar comparisons yielded a lower risk for pancreatic cancer for 2–3 years of exposure (RR 0.34, 95% CI 0.17, 0.66), and for liver cancer for 3–4 years (RR 0.36, 95% CI 0.14, 0.94) and >6 years (RR 0.22, 95% CI 0.05, 0.92). In addition, the results suggested an increase in the risk for bladder (RR 1.41, 95% CI 0.92, 2.17) and colorectal cancer (RR 1.28, 95% CI 0.94, 1.75) in men for <0.5 years and for breast cancer for <0.5 years (RR 1.32, 95% CI 0.98, 1.79) and 0.5–1 years (RR1.32, 95% CI 0.95, 1.85) in women.

In similar analyses performed using the 1 year categories for longer duration of exposure (≥6 years) and both sexes combined (not sex-specific cancers), results remained similar (ESM Fig. 1): RR (95% CI) 1.41 (1.11, 1.79) for colorectal cancer for <0.5 year, 0.67 (0.43, 1.03) for pancreatic cancer for 2–3 years, 0.44 (0.21, 0.91) for liver cancer for 3–4 years and 1.60 (1.05, 2.43) for melanoma of the skin for 4–5 years of insulin glargine vs human insulin use. Comparisons of insulin detemir vs human insulin and insulin glargine vs insulin detemir also showed no consistent differences in sex- and site-specific incidence rates (ESM Table 1) as well as in the analyses combining both sexes (ESM Figs 2 and 3).

**Fig. 3:**
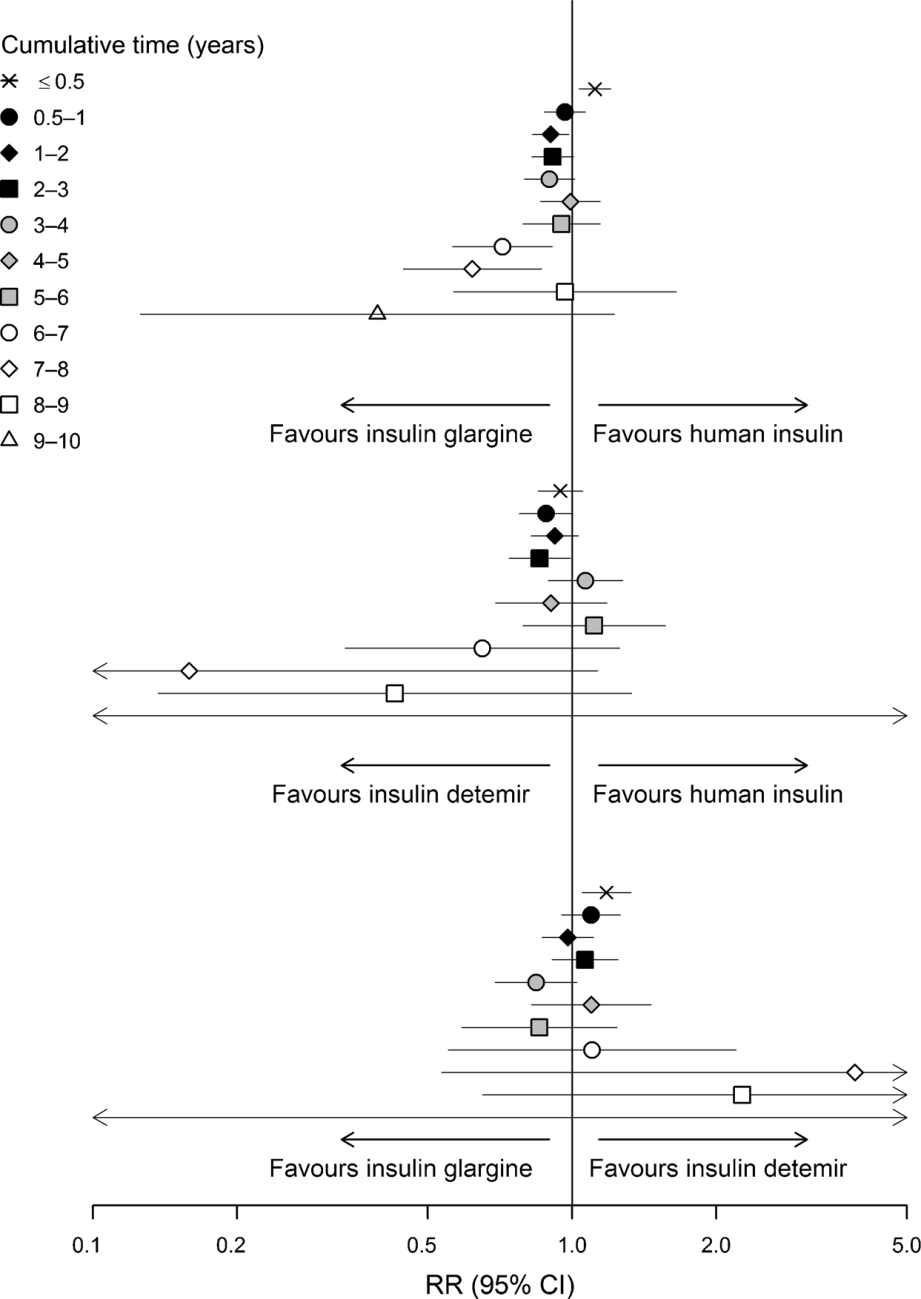
RR (adjusted for age, calendar time, sex, NIADs, duration of insulin-treated diabetes, country) with 95% CI for any cancer: pairwise comparisons of insulin glargine, insulin detemir and human insulin by the cumulative treatment time (years)

For any cancer in women, we found an elevated risk for 0.5 year of insulin glargine use relative to human insulin (RR 1.17, 95% CI 1.03, 1.32); in men, there was a lower risk for 0.5–1 year (RR 0.87, 95% CI 0.77, 1.00), 1–2 years (RR 0.84, 95% CI 0.73, 0.95) and >6 years (RR 0.61, 95% CI 0.48, 0.78) of exposure (Figs 1, 2 and ESM Table 1). Analysis performed without stratifying on sex (Fig. 3) yielded an elevated risk for any cancer for insulin glargine use relative to human insulin for 0.5 year (RR 1.12, 95% CI 1.03, 1.20) and a lower risk for 1–2 years (RR 0.90, 95% CI 0.83, 0.98), 6–7 years (RR 0.72, 95% CI 0.56, 0.91) and 7–8 years (RR 0.62, 95% CI 0.44, 0.86). Other analyses yielded a lower risk for any cancer in men for 0.5–1, 2–3 and >6 years of insulin detemir use relative to that of human insulin (ESM Table 1), and an increased risk in men and women combined (RR 1.18, 95% CI 1.05, 1.33) for <0.5 years of insulin glargine use relative to that of insulin detemir (Fig. 3).

Results were robust across a range of sensitivity analyses (ESM Tables 2, 3). Inclusion of the population with type 2 diabetes (1.31 million person-years [90%], 21,151cancer cases [99%]) yielded similar results to those of the primary analysis. The results did not change after either restriction of the study period to the time after insulin glargine’s launch (1.12 million person-years [77%], 16,838 cancer cases [79%]) or restriction of the study population to the Nordic cohorts. For breast and endometrial cancers, the results remained unchanged after further adjustment for menopausal status.

## Discussion

In the cohort study of 327,112 new insulin users from five European countries, we addressed the relationship between insulin use and cancer incidence of ten site-specific cancers and any cancer, when mitigating methodological shortcomings and biases involved in previous studies. Comparisons of cancer incidence by cumulative treatment time using active comparators showed no consistent differences in the cancer risk for insulin glargine or insulin detemir use relative to that of human insulin use. Although we observed increased and decreased cancer risks for some sites and treatment durations, no trends in the risk with duration of use were seen.

The findings of previous observational studies on the relationship between cancer risk and use of insulin glargine are conflicting and may involve methodological limitations and biases [15, 17]. Most of the current evidence is based on short follow-up and use of an elementary representation of exposure, ignoring the dose or duration of insulin exposure. The latter may result in the inadequate risk estimates, especially when treatment durations of widely differing lengths are considered equivalent [15, 19]. Of the recent studies with a short follow-up as a main limitation [15], only four were built on a new-user cohort [37–40], and two assessed cancer risk by treatment durations using an active-comparator approach [37, 38].

In the observational study on new users of insulin glargine (*n* = 43,306) and human insulin (*n* = 9147) enrolled in a US health plan, no association with the risk for prostate, breast, colon and any cancer was found for treatment durations of 0–6, 6–12, 12–24 or ≥24 months [38]. However, the risk estimates reported in this study were imprecise due to the small reference group. An observational study on a cohort of 70,027 new insulin users in France found no differences in risk at median follow-up <3 years for bladder, breast, colorectal, head and neck, liver, lung and kidney cancer between new users of insulin glargine and other basal insulins, when excluding the first year of use and defining exposure as ever-use or cumulative dose [40]. A study of a cohort of 19,337 incident insulin users from the Netherlands found a decreased risk for overall and colon cancer but no difference in risk for bladder, respiratory tract and prostate cancer, when comparing time-dependently defined cumulative time using insulin glargine to that using human insulin, though without further distinction between different treatment durations [39].

In the present study, we found that 22–63% of cancer cases were diagnosed within the first year after starting insulin treatment. Exclusion of 0.5–1 year of insulin use, or analysis of short-term use only, may preclude observing the actual dynamics of cancer incidence among insulin users and thus may hinder a better understanding of the nature of the link between diabetes and cancer. For the first half-year of cumulative treatment time on insulin glargine relative to that on human insulin, we found an increased risk for colorectal and any cancer in women and both sexes combined, and for endometrial cancer in women. These findings suggest possible involvement of detection or protopathic bias [41]. The latter is more likely to affect the present study, wherein use of insulin glargine was less common than that of human insulin and initiation of insulin glargine was often preceded by use of other insulins, predominantly human insulin. According to the current guidelines, switching from human insulin to insulin glargine should be considered if an individual has hypoglycaemia or fails to reach the target glucose level [42]. Poor glycaemic control may be a sign of underlying cancer and thus switching insulins because of highly variable blood glucose could be associated with more frequently detected cancer.

Although the results of the present study suggest a shift towards increased risk for breast cancer for the initial year of insulin glargine vs human insulin use, no differences were found for longer durations of treatment, when using appropriate comparators and adjusting for the overall time on insulin. In contrast to our study, three recent studies found an association between use of insulin glargine and increased risk for breast cancer [37, 39, 43]. However, those findings may reflect an imbalance in comparator and exposure characteristics rather than differences in cancer risk due to insulin use itself. A study on the UK’s General Practice Research Database cohort revealed an elevated risk for >5 years since the start of insulin glargine, when comparing insulin glargine users with previous use of insulin vs prevalent users of other insulins, matched on prior insulin duration at baseline [37]. Comparison by time since start of insulin glargine use is likely to provide an unbiased estimate for short-term use but not for longer use where the actual time on insulin and time since the initiation may differ noticeably between comparators. A study from the Netherlands reported an elevated risk for breast cancer when using cumulative durations without differentiation between them, thus ignoring an imbalance between comparators in the follow-up times (median of 2.2 years for insulin glargine users and 3.8 years for human insulin users) [39]. A recent study on a cohort of 12,468 new insulin users from the UK’s CPRD also reported an increased risk for breast cancer among new users of insulin glargine with extensive past exposure to other insulins, when comparing insulin glargine use of >3 years with ever-use of other insulins [43]. Comparison of specific duration with ever-use may yield a biased result, especially when the time period covered by specific duration differs considerably from the period covered by ever-use.

When comparing longer cumulative treatment time on insulin glargine with time on human insulin, we found a decreased risk for some cancers and treatment durations. However, the results for the other exposure categories showed no persistent differences even though an association between the increased cancer risk and use of insulin glargine would have been expected for the hypothesised effect. One possible explanation is that the better glycaemic control associated with use of insulin glargine, rather than human insulin [44], may play a part. So far, the evidence from epidemiological studies on the link between cancer risk and hyperglycaemia has been conflicting [45–48]. Similarly, increased site-specific cancer risks among individuals with type 1 and 2 diabetes, though showing a smaller excess risk for type 1 diabetes, suggest that a common diabetes-related determinant other than insulin use affects the cancer incidence [5]. Thus, to enhance knowledge on the interplay between diabetes and cancer, future research should focus on the effect of long-term glycaemic control itself, rather than different diabetes treatments.

This study has several strengths. To our knowledge, the present study is the first to employ a semi-aggregate level analysis across multiple populations from different countries to compare the effect of different insulin treatments on cancer risk. A semi-aggregate approach allowed us to analyse the study cohorts together using uniform methods. The size of the resulting cohort was fivefold that of the largest new-user cohort previously studied [40]. Our study had enough statistical power for the assessment of both sex- and site-specific cancer outcomes by cumulative durations; this assessment is considered by Renehan [16] to be an essential feature of an appropriately conducted pharmaco-epidemiological study on the link between insulin analogues and cancer risk.

Through the study design and analytical stages, we addressed important limitations and mitigated typical biases (Table 1) [15, 16, 24, 25, 49]. We adopted several other characteristics proposed by Renehan [16], including cohort design, use of validated data sources, exclusion of prevalent users and time-dependent definition of exposure. Moreover, we used an active-comparator design, which together with new-user design effectively reduces time-related biases and residual confounding [15, 25]. In addition, rather than censoring at switching or stopping an insulin, we followed diabetic individuals through the entire insulin prescribing span. The advantages listed above, along with use of nationwide (Nordic countries) and representative population-based (CPRD) cohorts, provide generalisable findings that can be directly applied to real-word decision making.

Our study does, however, have some limitations, including lack of information on important risk factors such as smoking, BMI, sedentary lifestyle, family history of cancer and diabetes duration, type, severity and comorbidities. Diabetes duration has been associated with changes in cancer risk [4, 49] and obesity was found to contribute to the increased risk of colorectal cancer among individuals with type 2 diabetes [50]. Studies comparing insulin glargine users with human insulin users found little evidence of confounding by diabetes duration, hospitalisations or cancer screening [40], or by BMI, smoking, income or HbA_1c_ levels [38, 43, 51]. In addition to the use of active comparators, which reduces residual confounding, we accounted for the duration of insulin-treated diabetes, which could be an effect modifier of the relationship between insulin glargine use and cancer risk [37, 43], and adjusted for baseline use of non-insulin glucose-lowering therapy which can be considered as a proxy for one’s diabetes stage when starting insulin treatment. We also performed sensitivity analyses to evaluate the effect of diabetes type.

Other disadvantages include lack of information on insulin dosage and the fact that exposure to non-insulin glucose-lowering therapy was defined as baseline usage without distinction between different therapies. Although we accounted for country-specific characteristics, we could not rule out any potential confounding effects resulting from the differences in insulin user profiles between the countries. In addition, examining numerous potential associations is likely to produce some false-positive results.

To summarise, the present multi-country study addressed the clinically important question of whether some of the commonly used insulin treatments should be preferred over others as being safer with respect to cancer risk. We found no persistent differences in the risk for ten specific cancers and any cancer, when comparing use of the insulin analogues insulin glargine or insulin detemir vs human insulin. These results add to the conclusive evidence on the absence of a relationship between cancer incidence and use of insulin analogues at follow-up exceeding 5 years. We see no indications to warrant withholding of the use of the insulin analogues insulin glargine and insulin detemir.

## Electronic supplementary material

ESM (PDF 1824 kb)

## Abbreviations

ATC: Anatomical Therapeutic Chemical
CARING: Cancer risk and insulin analogues
CPRD: Clinical Practice Research Datalink
DDD: Defined daily dose
HRT: Hormone replacement treatment
NIAD: Non-insulin antidiabetic drug
NSAID: Nonsteroidal anti-inflammatory drug
RR: Rate ratio
