# Supplementary material for: Cancer risk among insulin users: comparing analogues with human insulin in the CARING five-country cohort study
Source: Diabetologia. 2017 Jun 1;60(9):1691–703. doi: 10.1007/s00125-017-4312-5 (PMC5552833; doi:10.1007/s00125-017-4312-5)
Supplement: Supplementary file 1 — (PDF 1824 kb) [file 125_2017_4312_MOESM1_ESM.pdf]

Electronic supplementary material

ESM methods. Detailed description of the calculation of cumulative treatment time.

ESM Table 1. Adjusted rate ratios with 95% confidence interval for site- and sex-specific cancers: pairwise comparisons of glargine, detemir, and human insulin by the cumulative treatment time.

ESM Table 2. Results of sensitivity analyses, men.

ESM Table 3. Results of sensitivity analyses, women.

ESM Figure 1. Adjusted rate ratios for glargine versus human insulin.

ESM Figure 2. Adjusted rate ratios for detemir versus human insulin.

ESM Figure 3. Adjusted rate ratios for glargine versus detemir.

ESM Methods

Detailed description of the calculation of cumulative treatment time

Below we describe a time-dependent approach that we used to construct the cumulative exposure as a cumulative time on treatment. Hereafter, by exposure to specific insulin we mean a cumulative time on this insulin treatment. Prescription data form the Nordic registries included the date and amount purchased in defined daily doses (DDDs) [29], but no information on individual dosage (http://www.whocc.no/ddd/definition_and_general_considera/). For the CPRD cohort, we derived DDDs from the dosage information (substance strength and amount) contained by prescription data. Given a large effect of some individual's characteristics such as age, weight etc., on actual dosing, we assumed that cumulative time on particular insulin is more relevant exposure measure than actual DDDs per se. DDDs were used to calculate cumulative time under exposure, assuming a dose of 1 DDD per day. One DDD equals 40 IU for all insulins of interest (human insulin, insulin glargine and detemir), except human insulin for inhalation (A10AF01).

In exposure calculations we used the R language package **Epi** (Bendix Carstensen, Martyn Plummer, Esa Läärä, Michael Hills (2016). Epi: A Package for Statistical Analysis in Epidemiology. R package version 2.0. URL http://CRAN.R-project.org/package=Epi). More specifically, we used the **gen.exp** fuction, which is designed for construction of relevant covariates from purchase records. The information we used to construct the exposure variables included person identification numbers, date of each purchase, amount purchased, assumed daily dose, follow-up purchase (prescription) data. The basic ideas behind this function and illustrative examples are presented in the Tabulation outline (http://www.bendixcarstensen.com/DCRC/Tabulate/tabulate.pdf) by Bendix Carstensen.

For each insulin type of interest, the exposure was defined to begin on the date of first purchase (prescription), after which point an individual was considered exposed for the rest of his/her follow-up period. We generated exposure covariates for the insulin treatments of interest (human insulin, insulin glargine, insulin detemir) for the entire follow-up of each individual. We transformed each purchase (prescription) record into a period it covered according to the amount of DDDs, when assuming a constant dose rate of 1 DDD per day. For instance, 400 IUs of insulin glargine equals to ten DDDs and, thus, cover the period of ten days. Therefore, the period covered by this purchase (prescription) is a time from the date of purchase (prescription) to the date of purchase plus 10 days. All purchases (prescriptions) were translated into exposure time in the same way for all persons.

To record any changes in one's medication state during the follow-up period, we split the individual follow-up time into 120-day intervals, and updated the exposure at the beginning of each interval. In

the gen.exp function, use of information of dose rate was allowed by setting argument **use.dpt=TRUE**. In episodes of repeated prescriptions, this resulted in stacked exposure periods. By setting **push.max=1** we allowed the start of exposure to particular purchase to be pushed into the future by maximum of 1 day. Time on a particular insulin accumulated until exposure stopped, and cumulative exposure remained unchanged unless individual resumed treatment. In episodes of repeated prescriptions, possible gaps between the periods not covered by adjacent purchases were ignored in the calculation of cumulative treatment time.

Resulting datasets (one for each cohort) consisted of records with the intervals of short length, at the start for which cumulative treatment time was calculated for each of three insulin types of interest. Some of the individuals were treated with only one insulin type through the entire follow-up period, whereas others used different types of insulin. Thus, there were individuals who remained unexposed to all of the insulins of interest or to some of them, while for others the time at each of three insulin treatments of interest accumulated.

For instance, an individual with an entire follow-up length of four years started with human insulin and used it during the first three years of follow-up period so that purchased amount covered only 2.5 years. After two years he or she started detemir and during one year purchased amount corresponding to 1.08 years of exposure. Because the gaps of six months between the purchases of human insulin do not contribute to the cumulative treatment time, and because of quitting this treatment after three years, exposure to human insulin reached maximum of 2.5 years after three years and remained at this level until the end of follow-up. During the first two years of follow-up, individual remained unexposed to detemir and during the third year of follow-up the exposure exceeded one exposure year by one month so that this one month of the exposure was not accounted for at the end of follow-up. The exposure to human insulin remained zero throughout the entire follow-up.

Finally, three exposure variables (human insulin, insulin glargine, insulin detemir) were categorized into broader and finer exposure covariates by dividing cumulative treatment time into half-year categories for the first year, followed by one-year categories for longer exposure; the last categories were > 6 years for the broadly, and > 12 years for the finely categorized long-term exposure. In addition, each exposure variable incorporated a category to denote whether one stayed unexposed to the specific insulin represented by the variable.

ESM tables

ESM Table 1. Adjusted (all sites and any cancer: age, calendar time, NIADs, duration of insulin-treated diabetes, country; liver, colorectal, breast, and endometrial cancer: additional adjustment for relevant co-medications) rate ratios (RR) with 95% confidence interval (CI) for site- and sex-specific cancers: pairwise comparisons of glargine, detemir, and human insulin by the cumulative treatment time (years).

Cumulative treatment time, years	**Glargine versus Human Insulin**				**Detemir versus Human Insulin**				**Glargine versus Detemir**			
	Male		Female		Male		Female		Male		Female	
	RR (95% CI)	P-value	RR (95% CI)	P-value	RR (95% CI)	P-value	RR (95% CI)	P-value	RR (95% CI)	P-value	RR (95% CI)	P-value
**Liver cancer**												
0-0.5	0.89 (0.54, 1.47)	.64	1.10 (0.49, 2.48)	.83	1.01 (0.54, 1.88)	.97	0.66 (0.19, 2.33)	.52	0.88 (0.44, 1.76)	0.72	1.66 (0.45, 6.16)	.45
0.5-1	0.89 (0.51, 1.56)	.69	0.47 (0.14, 1.52)	.21	1.35 (0.74, 2.44)	.33	0.42 (0.09, 1.96)	.27	0.66 (0.34, 1.28)	0.22	1.12 (0.20, 6.21)	.90
1-2	1.11 (0.69, 1.77)	.68	0.58 (0.19, 1.76)	.34	0.75 (0.38, 1.47)	.40	0.90 (0.27, 3.02)	.87	1.48 (0.74, 2.98)	0.27	0.64 (0.17, 2.46)	.52
2-3	1.13 (0.66, 1.96)	.66	0.62 (0.20, 1.86)	.39	0.78 (0.33, 1.89)	.59	0.57 (0.12, 2.72)	.48	1.44 (0.58, 3.60)	0.43	1.07 (0.2, 5.62)	.93
3-4	0.36 (0.14, 0.94)	.04	0.52 (0.16, 1.66)	.27	1.71 (0.77, 3.79)	.19	0.76 (0.16, 3.55)	.73	0.21 (0.07, 0.65)	0.01	0.68 (0.12, 3.79)	.66
4-5	1.11 (0.53, 2.31)	.78	0.74 (0.20, 2.78)	.66	0.92 (0.21, 3.90)	.91	NE	.98	1.21 (0.26, 5.57)	0.80	NE	.98
5-6	0.75 (0.28, 1.96)	.55	NE	.98	NE	.97	NE	.99	NE	0.97	0.56 (NE)	.99
>6	0.22 (0.05, 0.92)	.04	0.56 (0.12, 2.56)	.45	NE	.98	NE	.99	NE	0.98	NE	.99
**Pancreatic cancer**												
0-0.5	1.08 (0.86, 1.36)	.52	0.85 (0.64, 1.13)	.25	0.76 (0.54, 1.07)	.11	0.67 (0.44, 1.02)	.06	1.42 (0.99, 2.03)	.05	1.26 (0.8, 1.98)	.32
0.5-1	0.9 (0.61, 1.34)	.60	1.02 (0.67, 1.54)	.94	0.71 (0.40, 1.26)	.25	0.84 (0.46, 1.51)	.56	1.26 (0.67, 2.35)	.47	1.22 (0.64, 2.33)	.55
1-2	0.67 (0.44, 1.03)	.07	0.99 (0.64, 1.51)	.95	0.87 (0.52, 1.44)	.58	1.51 (0.93, 2.47)	.10	0.77 (0.43, 1.39)	.39	0.65 (0.37, 1.14)	.13
2-3	0.34	.002	1.32	.36	0.39	.05	0.85	.76	0.86	.78	1.55	.43

Cumulative treatment time, years	Glargine versus Human Insulin				Detemir versus Human Insulin				Glargine versus Detemir			
	Male		Female		Male		Female		Male		Female	
	RR (95% CI)	P-value	RR (95% CI)	P-value	RR (95% CI)	P-value	RR (95% CI)	P-value	RR (95% CI)	P-value	RR (95% CI)	P-value
	(0.17, 0.66)		(0.73, 2.41)		(0.16, 0.99)		(0.30, 2.42)		(0.29, 2.52)		(0.52, 4.67)	
3-4	0.53 (0.26, 1.10)	.09	0.76 (0.31, 1.85)	.54	1.07 (0.45, 2.52)	.89	NE	.96	0.50 (0.18, 1.41)	.19	NE	.96
4-5	1.16 (0.52, 2.59)	.72	1.29 (0.51, 3.23)	.59	0.56 (0.08, 4.17)	.57	1.56 (0.36, 6.75)	.55	2.07 (0.26, 16.57)	.49	0.83 (0.17, 4.12)	.82
5-6	1.13 (0.45, 2.85)	.79	0.62 (0.14, 2.68)	.52	1.71 (0.39, 7.47)	.47	NE	.98	0.66 (0.13, 3.30)	.61	NE	.98
>6	NE	.94	1.01 (0.31, 3.32)	.98	NE	.98	2.51 (0.34, 18.62)	.37	0.63 (NE)	.99	0.40 (0.04, 3.91)	.43
**Lung cancer**												
0-0.5	1.10 (0.81, 1.49)	.54	1.27 (0.83, 1.96)	.03	1.17 (0.80, 1.71)	.41	1.30 (0.77, 2.21)	.33	0.94 (0.62, 1.43)	.77	0.98 (0.54, 1.78)	.94
0.5-1	0.92 (0.62, 1.37)	.67	1.21 (0.67, 2.18)	.53	0.79 (0.47, 1.33)	.38	0.90 (0.41, 1.96)	.79	1.16 (0.65, 2.05)	.62	1.35 (0.57, 3.17)	.49
1-2	1.20 (0.87, 1.63)	.26	0.84 (0.52, 1.37)	.49	0.78 (0.49, 1.22)	.28	0.42 (0.19, 0.93)	.03	1.54 (0.96, 2.47)	.07	2.01 (0.86, 4.73)	.11
2-3	0.70 (0.47, 1.06)	.09	0.75 (0.40, 1.40)	.36	0.64 (0.35, 1.17)	.15	1.07 (0.52, 2.24)	.85	1.10 (0.56, 2.15)	.78	0.7 (0.29, 1.64)	.41
3-4	0.88 (0.56, 1.4)	.59	1.08 (0.52, 2.23)	.83	1.11 (0.57, 2.15)	.76	2.05 (0.92, 4.57)	.08	0.80 (0.38, 1.67)	.54	0.53 (0.21, 1.35)	.18
4-5	1.30 (0.76, 2.21)	.34	2.06 (0.99, 4.28)	.05	NE	.95	NE	.96	NE	.95	NE	.95
5-6	1.11 (0.54, 2.28)	.77	1.66 (0.71, 3.87)	.24	1.16 (0.28, 4.83)	.83	NE	.97	0.95 (0.21, 4.43)	.95	NE	.97
>6	0.56 (0.28, 1.11)	.10	0.46 (0.14, 1.49)	.19	NE	.97	0.74 (0.10, 5.48)	.77	NE	.98	0.61 (0.06, 5.93)	.67
**Melanoma of skin**												
0-0.5	1.07 (0.63, 1.81)	.80	1.62 (0.91, 2.89)	.10	0.93 (0.45, 1.93)	.85	1.73 (0.85, 3.51)	.13	1.15 (0.52, 2.53)	.73	0.94 (0.44, 2.00)	.87
0.5-1	0.66 (0.36, 1.22)	.18	1.17 (0.60, 2.28)	.65	0.67 (0.30, 1.46)	.31	0.67 (0.25, 1.80)	.43	0.99 (0.42, 2.35)	.98	1.74 (0.62, 4.87)	.29
1-2	1.16 (0.72, 1.87)	.54	1.22 (0.68, 2.17)	.51	1.24 (0.68, 2.25)	.48	1.03 (0.48, 2.24)	.93	0.94 (0.51, 1.73)	.84	1.18 (0.53, 2.62)	.69
2-3	0.82 (0.46, 1.46)	.50	1.92 (1.02, 3.61)	.04	1.01 (0.46, 2.19)	.99	0.99 (0.33, 2.94)	.99	0.81 (0.35, 1.89)	.63	1.93 (0.65, 5.75)	.24

Cumulative treatment time, years	Glargine versus Human Insulin				Detemir versus Human Insulin				Glargine versus Detemir			
	Male		Female		Male		Female		Male		Female	
	RR (95% CI)	P-value	RR (95% CI)	P-value	RR (95% CI)	P-value	RR (95% CI)	P-value	RR (95% CI)	P-value	RR (95% CI)	P-value
3-4	1.15 (0.63, 2.12)	.65	0.72 (0.29, 1.79)	.48	0.72 (0.22, 2.38)	.59	0.37 (0.05, 2.8)	.34	1.60 (0.47, 5.52)	.46	1.93 (0.23, 16.10)	.55
4-5	0.89 (0.42, 1.86)	.75	3.55 (1.68, 7.47)	.001	2.91 (1.20, 7.08)	.02	3.44 (0.98, 12.08)	.05	0.30 (0.11, 0.86)	.03	1.03 (0.29, 3.66)	.96
5-6	1.58 (0.52, 4.8)	.42	1.52 (0.51, 4.60)	.45	NE	.97	1.90 (0.25, 14.62)	.55	NE	.97	0.80 (0.09, 7.27)	.85
>6	1.25 (0.58, 2.69)	.57	1.76 (0.66, 4.68)	.26	NE	.98	NE	.97	NE	.98	NE	.97
**Bladder cancer**												
0-0.5	1.41 (0.92, 2.17)	.12	1.44 (0.61, 3.37)	.41	1.37 (0.76, 2.46)	.30	0.88 (0.20, 3.89)	.87	1.03 (0.54, 1.95)	.93	1.63 (0.34, 7.82)	.54
0.5-1	0.97 (0.56, 1.65)	.90	2.91 (0.89, 9.48)	.08	0.9 (0.43, 1.86)	.77	2.01 (0.39, 10.48)	.41	1.08 (0.49, 2.38)	.86	1.45 (0.29, 7.33)	.66
1-2	0.75 (0.46, 1.24)	.27	0.84 (0.29, 2.45)	.75	1.36 (0.79, 2.35)	.27	1.10 (0.30, 4.08)	.88	0.55 (0.29, 1.04)	.07	0.76 (0.18, 3.31)	.72
2-3	0.93 (0.56, 1.52)	.76	0.64 (0.21, 1.99)	.44	0.96 (0.47, 1.98)	.92	0.45 (0.06, 3.53)	.45	0.96 (0.44, 2.09)	.92	1.42 (0.16, 12.9)	.76
3-4	0.92 (0.52, 1.64)	.78	0.75 (0.16, 3.56)	.72	1.37 (0.61, 3.06)	.45	1.27 (0.16, 10.31)	.82	0.67 (0.28, 1.64)	.38	0.59 (0.05, 6.65)	.67
4-5	1.67 (0.86, 3.23)	.13	1.19 (0.25, 5.74)	.82	1.06 (0.25, 4.49)	.93	NE	.98	1.57 (0.35, 6.96)	.56	NE	.98
5-6	0.64 (0.19, 2.15)	.47	1.91 (0.40, 9.16)	.42	NE	.97	NE	.99	NE	.97	NE	.99
>6	0.57 (0.18, 1.86)	.35	0.69 (0.09, 5.40)	.73	NE	.98	NE	.99	NE	.98	NE	.99
**Colorectal cancer**												
0-0.5	1.28 (0.94, 1.75)	.12	1.54 (1.06, 2.25)	0.03	0.89 (0.55, 1.43)	0.64	0.87 (0.47, 1.61)	.65	1.44 (0.86, 2.41)	.17	1.78 (0.93, 3.41)	.08
0.5-1	0.84 (0.56, 1.26)	.40	0.80 (0.46, 1.40)	0.44	0.76 (0.45, 1.29)	0.32	1.81 (1.07, 3.08)	.03	1.10 (0.61, 1.99)	.74	0.44 (0.23, 0.85)	.01
1-2	0.78 (0.56, 1.08)	.14	1.05 (0.69, 1.60)	0.84	0.73 (0.47, 1.13)	0.16	0.64 (0.32, 1.26)	.20	1.07 (0.66, 1.74)	.79	1.64 (0.8, 3.35)	.18
2-3	1.06 (0.75, 1.50)	.72	1.18 (0.74, 1.90)	0.49	0.93 (0.56, 1.54)	0.77	0.81 (0.36, 1.80)	.60	1.15 (0.67, 1.98)	.62	1.46 (0.63, 3.39)	.38

Cumulative treatment time, years	Glargine versus Human Insulin				Detemir versus Human Insulin				Glargine versus Detemir			
	Male		Female		Male		Female		Male		Female	
	RR (95% CI)	P-value	RR (95% CI)	P-value	RR (95% CI)	P-value	RR (95% CI)	P-value	RR (95% CI)	P-value	RR (95% CI)	P-value
3-4	0.89 (0.56, 1.41)	.62	0.63 (0.31, 1.29)	0.21	1.01 (0.51, 2.03)	0.97	1.75 (0.82, 3.76)	.15	0.88 (0.41, 1.89)	.74	0.36 (0.14, 0.94)	.04
4-5	1.03 (0.59, 1.80)	.92	1.15 (0.57, 2.31)	0.70	1.12 (0.45, 2.82)	0.80	1.82 (0.64, 5.14)	.26	0.92 (0.33, 2.51)	.86	0.63 (0.20, 2.03)	.44
5-6	0.69 (0.29, 1.62)	.39	0.78 (0.28, 2.20)	0.64	0.96 (0.23, 3.99)	0.96	2.64 (0.80, 8.71)	.11	0.72 (0.14, 3.56)	.68	0.30 (0.07, 1.33)	.11
>6	0.50 (0.23, 1.08)	.08	0.59 (0.21, 1.62)	0.30	NE	0.96	NE	.95	NE	.96	NE	.95
**Non-Hodgkin's lymphoma**												
0-0.5	0.70 (0.36, 1.35)	.29	0.68 (0.33, 1.42)	.307	0.49 (0.17, 1.40)	0.183	0.86 (0.35, 2.10)	.73	1.42 (0.46, 4.45)	.54	0.80 (0.29, 2.24)	.67
0.5-1	0.93 (0.44, 1.94)	.84	1.28 (0.55, 2.98)	.570	1.14 (0.48, 2.73)	0.766	1.15 (0.40, 3.31)	.80	0.81 (0.31, 2.12)	.67	1.11 (0.37, 3.33)	.85
1-2	0.96 (0.46, 2.01)	.91	1.00 (0.47, 2.15)	.99	2.00 (0.94, 4.25)	.07	1.34 (0.54, 3.28)	.53	0.48 (0.21, 1.12)	.09	0.75 (0.28, 1.98)	.56
2-3	0.59 (0.23, 1.47)	.25	1.28 (0.59, 2.77)	.54	2.02 (0.87, 4.69)	.10	1.17 (0.38, 3.56)	.79	0.29 (0.10, 0.84)	.02	1.09 (0.34, 3.47)	.88
3-4	1.16 (0.55, 2.44)	.69	0.80 (0.26, 2.49)	.70	NE	.97	1.25 (0.28, 5.65)	.77	NE	.97	0.64 (0.12, 3.53)	.61
4-5	1.33 (0.55, 3.22)	.53	1.04 (0.33, 3.24)	.94	NE	.98	NE	.97	NE	.98	NE	.97
5-6	0.85 (0.19, 3.80)	.84	1.43 (0.29, 7.08)	.66	NE	.99	3.07 (0.36, 25.99)	.30	NE	.99	0.47 (0.04, 5.20)	.54
>6	1.43 (0.54, 3.84)	.47	0.28 (0.04, 2.16)	.22	NE	.99	NE	.99	NE	.99	NE	.99
**Prostate cancer**												
0-0.5	1.08 (0.87, 1.35)	.48			0.96 (0.71, 1.31)	.81			1.12 (0.80, 1.58)	.50		
0.5-1	0.93 (0.71, 1.21)	.59			0.84 (0.59, 1.20)	.34			1.10 (0.75, 1.63)	.62		
1-2	1.00 (0.79, 1.27)	.99			0.96 (0.70, 1.32)	.81			1.04 (0.75, 1.45)	.82		
2-3	0.90 (0.69, 1.18)	.49			0.87 (0.59, 1.29)	.50			1.03 (0.68, 1.58)	.88		

Cumulative treatment time, years	Glargine versus Human Insulin				Detemir versus Human Insulin				Glargine versus Detemir			
	Male		Female		Male		Female		Male		Female	
	RR (95% CI)	P-value	RR (95% CI)	P-value	RR (95% CI)	P-value	RR (95% CI)	P-value	RR (95% CI)	P-value	RR (95% CI)	P-value
3-4	0.98 (0.71, 1.34)	.88			1.3 (0.82, 2.04)	.26			0.75 (0.46, 1.24)	.26		
4-5	0.86 (0.57, 1.29)	.45			0.55 (0.20, 1.48)	.23			1.57 (0.55, 4.47)	.40		
5-6	0.91 (0.55, 1.50)	.70			1.30 (0.48, 3.54)	.61			0.70 (0.24, 2.07)	.52		
>6	0.81 (0.51, 1.31)	.40			0.86 (0.21, 3.50)	.84			0.94 (0.22, 4.06)	.94		
**Breast cancer**												
0-0.5			1.32 (0.98, 1.79)	.07			0.93 (0.59, 1.45)	.74			1.43 (0.88, 2.32)	.15
0.5-1			1.32 (0.95, 1.85)	.10			1.04 (0.66, 1.61)	.88			1.28 (0.80, 2.05)	.31
1-2			1.18 (0.88, 1.58)	.27			1.24 (0.86, 1.78)	.26			0.95 (0.64, 1.41)	.81
2-3			1.08 (0.78, 1.50)	.65			0.93 (0.58, 1.51)	.78			1.16 (0.69, 1.95)	.58
3-4			0.86 (0.57, 1.30)	.48			0.90 (0.48, 1.70)	.75			0.95 (0.47, 1.91)	.89
4-5			1.05 (0.65, 1.68)	.86			1.69 (0.89, 3.19)	.11			0.62 (0.30, 1.28)	.20
5-6			0.96 (0.52, 1.78)	.90			1.46 (0.59, 3.66)	.42			0.66 (0.23, 1.87)	.43
>6			1.10 (0.66, 1.84)	.71			0.37 (0.05, 2.69)	.33			2.95 (0.39, 22.21)	.29
**Endometrial cancer**												
0-0.5			1.78 (1.07, 2.94)	.03			1.18 (0.57, 2.45)	.66			1.51 (0.69, 3.27)	.30
0.5-1			0.86 (0.49, 1.50)	.60			0.44 (0.18, 1.04)	.06			1.97 (0.78, 4.99)	.15
1-2			0.61 (0.35, 1.05)	.07			0.41 (0.18, 0.91)	.03			1.50 (0.62, 3.60)	.37
2-3			0.89 (0.49, 1.60)	.70			1.13 (0.54, 2.36)	.75			0.79 (0.35, 1.80)	.58
3-4			1.63	.11			1.35	.54			1.21	.71

Cumulative treatment time, years	Glargine versus Human Insulin				Detemir versus Human Insulin				Glargine versus Detemir			
	Male		Female		Male		Female		Male		Female	
	RR (95% CI)	P-value	RR (95% CI)	P-value	RR (95% CI)	P-value	RR (95% CI)	P-value	RR (95% CI)	P-value	RR (95% CI)	P-value
			(0.89, 2.96)				(0.52, 3.51)				(0.44, 3.30)	
4-5			0.98 (0.45, 2.15)	.96			0.88 (0.21, 3.71)	.86			1.12 (0.24, 5.28)	.89
5-6			0.70 (0.24, 2.00)	.51			NE	.95			NE	.95
>6			0.34 (0.08, 1.42)	.14			2.68 (0.63, 11.42)	.18			0.13 (0.02, 0.92)	.04
**Any cancer** [a]												
0-0.5	1.09 (0.99, 1.20)	.08	1.17 (1.03, 1.32)	.01	0.88 (0.76, 1.01)	.08	1.05 (0.89, 1.24)	.54	1.24 (1.06, 1.45)	.01	1.11 (0.92, 1.33)	.27
0.5-1	0.87 (0.77, 1.00)	.04	1.12 (0.96, 1.30)	.15	0.83 (0.70, 0.98)	.03	0.97 (0.79, 1.18)	.73	1.06 (0.88, 1.27)	.55	1.16 (0.93, 1.44)	.18
1-2	0.90 (0.80, 1.01)	.06	0.90 (0.79, 1.04)	.16	0.89 (0.77, 1.03)	.13	0.95 (0.8, 1.14)	.60	1.01 (0.86, 1.18)	.93	0.95 (0.78, 1.15)	.59
2-3	0.84 (0.73, 0.95)	.007	1.04 (0.89, 1.22)	.65	0.79 (0.65, 0.96)	.02	0.96 (0.76, 1.21)	.71	1.06 (0.86, 1.30)	.59	1.08 (0.85, 1.39)	.52
3-4	0.91 (0.78, 1.06)	.23	0.88 (0.72, 1.07)	.20	1.07 (0.85, 1.35)	.56	1.06 (0.79, 1.4)	.71	0.85 (0.66, 1.09)	.20	0.83 (0.61, 1.14)	.26
4-5	0.93 (0.77, 1.12)	.46	1.10 (0.88, 1.37)	.40	0.88 (0.62, 1.25)	.48	0.94 (0.62, 1.41)	.76	1.06 (0.72, 1.55)	.77	1.17 (0.76, 1.82)	.48
5-6	0.96 (0.75, 1.22)	.72	0.95 (0.71, 1.27)	.75	0.95 (0.57, 1.57)	.84	1.29 (0.81, 2.06)	.29	1.01 (0.59, 1.73)	.98	0.74 (0.44, 1.25)	.26
>6	0.61 (0.48, 0.78)	<0.001	0.76 (0.58, 1.01)	.06	0.18 (0.06, 0.55)	.003	0.84 (0.45, 1.58)	.59	3.46 (1.09, 10.98)	.04	0.91 (0.46, 1.78)	.78

[a] Any cancer except non-melanoma skin cancer

Abbreviations: RR – rate ratio, CI – confidence interval, NE – not estimable.

ESM Table 2. Results of the sensitivity analyses for men: adjusted (all sites and any cancer: age, calendar time, NIADs, duration of insulin-treated diabetes, country; liver, colorectal, breast, and endometrial cancer: additional adjustment for relevant co-medications) rate ratios (RR) with 95% confidence interval (CI) for site-specific cancers and any cancer. Pairwise comparisons of glargine, detemir, and human insulin by the cumulative treatment time (years), when restricting the study population (type 2 diabetes, or Nordic countries, or study entry in 2000 or later).

Cumulative treatment time, years	Type 2 diabetes			Nordic population			Entry from 2000 onward		
	RR	95% CI	P	RR	95% CI	P	RR	95% CI	P
**Liver cancer**									
**Glargine vs Human insulin**									
0-0.5	0.89	0.54, 1.48	0.661	0.91	0.54, 1.54	0.718	0.87	0.51, 1.49	0.603
0.5-1	0.90	0.52, 1.56	0.705	0.86	0.48, 1.55	0.620	0.65	0.33, 1.29	0.222
1-2	1.09	0.68, 1.75	0.708	0.89	0.53, 1.51	0.673	0.78	0.44, 1.38	0.392
2-3	1.14	0.66, 1.97	0.643	1.01	0.55, 1.84	0.985	1.35	0.71, 2.56	0.364
3-4	0.37	0.14, 0.96	0.040	0.40	0.15, 1.05	0.061	0.37	0.13, 1.06	0.064
4-5	1.25	0.61, 2.53	0.543	1.27	0.58, 2.76	0.548	0.91	0.36, 2.27	0.839
5-6	0.76	0.29, 1.99	0.574	0.94	0.35, 2.48	0.895	1.10	0.37, 3.26	0.865
>6	0.23	0.05, 0.95	0.042	0.16	0.02, 1.19	0.074	0.24	0.03, 1.74	0.157
**Glargine vs Detemir**									
0-0.5	0.88	0.44, 1.76	0.715	1.34	0.58, 3.07	0.489	1.25	0.54, 2.88	0.603
0.5-1	0.66	0.34, 1.28	0.221	0.76	0.37, 1.55	0.447	0.53	0.24, 1.17	0.116
1-2	1.48	0.74, 2.99	0.268	1.20	0.58, 2.49	0.621	0.86	0.40, 1.85	0.699
2-3	1.44	0.58, 3.59	0.434	1.45	0.53, 3.95	0.471	1.64	0.54, 4.93	0.381
3-4	0.21	0.07, 0.65	0.007	0.24	0.07, 0.75	0.014	0.42	0.09, 1.88	0.257
4-5	1.33	0.29, 6.04	0.710	0.95	0.20, 4.40	0.944	0.59	0.12, 2.95	0.523
5-6	NE	NE	0.971	NE	NE	0.967	NE	NE	0.971
>6	NE	NE	0.980	NE	NE	0.988	NE	NE	0.990
**Detemir vs Human Insulin**									
0-0.5	1.02	0.55, 1.89	0.956	0.68	0.32, 1.45	0.316	0.69	0.32, 1.49	0.349
0.5-1	1.36	0.75, 2.46	0.317	1.14	0.59, 2.17	0.700	1.24	0.63, 2.43	0.531
1-2	0.74	0.37, 1.45	0.377	0.74	0.37, 1.49	0.401	0.90	0.45, 1.82	0.776
2-3	0.79	0.33, 1.90	0.600	0.70	0.27, 1.82	0.459	0.82	0.28, 2.41	0.722
3-4	1.75	0.79, 3.89	0.168	1.69	0.72, 3.98	0.227	0.87	0.26, 2.95	0.825
4-5	0.94	0.22, 3.98	0.928	1.34	0.31, 5.77	0.693	1.54	0.35, 6.65	0.567
5-6	0.00	NE	0.970	0.00	NE	0.967	0.00	NE	0.971
>6	0.00	NE	0.978	0.00	NE	0.986	0.00	NE	0.989
**Pancreatic cancer**									
**Glargine vs Human insulin**									
0-0.5	1.08	0.86, 1.35	0.534	1.01	0.79, 1.30	0.929	1.05	0.81, 1.35	0.712
0.5-1	0.91	0.61, 1.35	0.631	0.83	0.54, 1.27	0.392	0.82	0.53, 1.27	0.373
1-2	0.67	0.44, 1.04	0.072	0.56	0.35, 0.91	0.019	0.54	0.32, 0.91	0.020
2-3	0.34	0.17, 0.67	0.002	0.32	0.16, 0.66	0.002	0.28	0.13, 0.63	0.002
3-4	0.54	0.26, 1.11	0.094	0.54	0.25, 1.17	0.118	0.61	0.27, 1.40	0.246
4-5	1.19	0.53, 2.65	0.675	0.68	0.23, 1.98	0.477	0.72	0.25, 2.13	0.557

Cumulative treatment time, years	Type 2 diabetes			Nordic population			Entry from 2000 onward		
	RR	95% CI	P	RR	95% CI	P	RR	95% CI	P
5-6	1.21	0.48, 3.06	0.681	1.33	0.48, 3.64	0.580	1.57	0.57, 4.35	0.381
>6	0.00	NE	0.967	0.00	NE	0.974	0.00	NE	0.977
**Glargine vs Detemir**									
0-0.5	1.41	0.99, 2.02	0.060	1.39	0.94, 2.05	0.096	1.43	0.96, 2.12	0.075
0.5-1	1.26	0.68, 2.35	0.468	1.17	0.61, 2.26	0.639	1.34	0.66, 2.70	0.419
1-2	0.77	0.43, 1.39	0.393	0.72	0.37, 1.38	0.316	0.66	0.33, 1.30	0.226
2-3	0.85	0.29, 2.50	0.771	0.78	0.26, 2.33	0.653	0.75	0.22, 2.57	0.648
3-4	0.50	0.18, 1.40	0.184	0.43	0.15, 1.24	0.117	0.44	0.14, 1.40	0.164
4-5	2.02	0.25, 16.20	0.507	NE	NE	0.978	NE	NE	0.980
5-6	1.28	0.15, 10.66	0.821	NE	NE	0.986	NE	NE	0.987
>6	0.62	NE	1.000	0.27	NE	0.999	0.28	NE	1.000
**Detemir vs Human Insulin**									
0-0.5	0.76	0.54, 1.07	0.119	0.73	0.50, 1.05	0.089	0.73	0.51, 1.07	0.104
0.5-1	0.72	0.41, 1.27	0.259	0.71	0.39, 1.28	0.255	0.61	0.32, 1.16	0.134
1-2	0.87	0.52, 1.45	0.596	0.78	0.45, 1.36	0.388	0.83	0.47, 1.47	0.517
2-3	0.40	0.16, 1.00	0.050	0.42	0.17, 1.05	0.064	0.37	0.13, 1.05	0.062
3-4	1.09	0.46, 2.58	0.845	1.27	0.53, 3.04	0.591	1.39	0.53, 3.62	0.503
4-5	0.59	0.08, 4.37	0.603	0.00	NE	0.978	0.00	NE	0.979
5-6	0.95	0.13, 7.19	0.961	0.00	NE	0.986	0.00	NE	0.988
>6	0.00	NE	0.986	0.00	NE	0.994	0.00	NE	0.996
**Lung cancer**									
**Glargine vs Human insulin**									
0-0.5	1.09	0.80, 1.47	0.593	1.06	0.76, 1.47	0.753	0.97	0.68, 1.38	0.865
0.5-1	0.92	0.62, 1.37	0.683	0.94	0.61, 1.46	0.779	0.98	0.61, 1.57	0.922
1-2	1.18	0.86, 1.62	0.295	1.09	0.76, 1.56	0.628	1.05	0.71, 1.55	0.797
2-3	0.71	0.47, 1.07	0.102	0.77	0.49, 1.20	0.242	0.73	0.45, 1.18	0.198
3-4	0.89	0.56, 1.42	0.631	0.94	0.57, 1.56	0.823	0.81	0.46, 1.41	0.456
4-5	1.38	0.82, 2.32	0.226	1.00	0.51, 1.98	0.991	0.89	0.42, 1.91	0.771
5-6	1.00	0.47, 2.12	0.992	0.89	0.35, 2.27	0.813	1.34	0.51, 3.48	0.550
>6	0.57	0.29, 1.13	0.108	0.62	0.25, 1.54	0.303	0.77	0.31, 1.93	0.584
**Glargine vs Detemir**									
0-0.5	0.92	0.60, 1.41	0.708	0.81	0.52, 1.27	0.352	0.76	0.48, 1.22	0.256
0.5-1	1.16	0.65, 2.05	0.620	1.16	0.62, 2.19	0.636	0.99	0.52, 1.88	0.964
1-2	1.52	0.94, 2.43	0.085	1.24	0.74, 2.07	0.407	1.12	0.65, 1.93	0.677
2-3	1.10	0.56, 2.14	0.788	1.01	0.50, 2.06	0.968	1.07	0.49, 2.31	0.873
3-4	0.99	0.44, 2.20	0.976	1.04	0.42, 2.60	0.931	1.60	0.47, 5.50	0.456
4-5	NE	NE	0.933	NE	NE	0.947	0.00	NE	0.952
5-6	0.84	0.18, 3.96	0.824	NE	NE	0.967	0.00	NE	0.971
>6	NE	NE	0.966	NE	NE	0.987	0.00	NE	0.989
**Detemir vs Human Insulin**									
0-0.5	1.18	0.81, 1.72	0.396	1.31	0.88, 1.93	0.184	1.27	0.85, 1.91	0.247
0.5-1	0.80	0.47, 1.34	0.390	0.81	0.46, 1.42	0.458	0.99	0.56, 1.77	0.976

Cumulative treatment time, years	Type 2 diabetes			Nordic population			Entry from 2000 onward		
	RR	95% CI	P	RR	95% CI	P	RR	95% CI	P
1-2	0.78	0.50, 1.23	0.284	0.88	0.54, 1.42	0.601	0.94	0.57, 1.55	0.802
2-3	0.65	0.35, 1.19	0.162	0.75	0.40, 1.43	0.388	0.69	0.34, 1.39	0.294
3-4	0.90	0.43, 1.89	0.789	0.91	0.39, 2.10	0.820	0.51	0.16, 1.62	0.252
4-5	0.00	NE	0.934	0.00	NE	0.947	0.00	NE	0.951
5-6	1.19	0.29, 4.93	0.813	0.00	NE	0.967	0.00	NE	0.971
>6	0.00	NE	0.965	0.00	NE	0.986	0.00	NE	0.989
**Melanoma of skin**									
**Glargine vs Human insulin**									
0-0.5	1.08	0.64, 1.83	0.774	1.05	0.60, 1.85	0.868	1.06	0.58, 1.95	0.843
0.5-1	0.66	0.36, 1.22	0.185	0.63	0.32, 1.24	0.177	0.72	0.35, 1.48	0.368
1-2	1.15	0.71, 1.87	0.565	1.12	0.67, 1.87	0.677	1.22	0.71, 2.12	0.468
2-3	0.79	0.44, 1.44	0.447	0.79	0.42, 1.49	0.465	0.71	0.35, 1.43	0.334
3-4	1.23	0.67, 2.27	0.503	1.37	0.72, 2.60	0.337	1.66	0.83, 3.30	0.150
4-5	0.81	0.37, 1.76	0.590	0.71	0.29, 1.71	0.443	0.44	0.13, 1.48	0.184
5-6	1.63	0.54, 4.95	0.388	1.59	0.45, 5.58	0.468	2.32	0.62, 8.72	0.212
>6	1.29	0.60, 2.79	0.520	1.46	0.61, 3.53	0.396	0.90	0.27, 3.02	0.869
**Glargine vs Detemir**									
0-0.5	1.14	0.52, 2.52	0.737	1.12	0.48, 2.60	0.788	1.00	0.42, 2.36	0.997
0.5-1	0.98	0.41, 2.34	0.971	0.92	0.36, 2.35	0.855	0.83	0.32, 2.16	0.703
1-2	0.90	0.49, 1.67	0.745	0.85	0.44, 1.62	0.621	0.97	0.48, 1.97	0.939
2-3	0.76	0.32, 1.78	0.521	0.67	0.28, 1.60	0.363	0.58	0.23, 1.52	0.269
3-4	1.58	0.46, 5.43	0.469	1.45	0.42, 5.02	0.559	1.31	0.38, 4.57	0.672
4-5	0.26	0.09, 0.76	0.014	0.24	0.07, 0.85	0.028	0.12	0.03, 0.52	0.005
5-6	NE	NE	0.981	NE	NE	0.986	NE	NE	0.988
>6	NE	NE	0.986	NE	NE	0.994	NE	NE	0.996
**Detemir vs Human Insulin**									
0-0.5	0.94	0.46, 1.96	0.876	0.93	0.43, 2.03	0.864	1.06	0.48, 2.33	0.882
0.5-1	0.67	0.31, 1.47	0.317	0.68	0.30, 1.58	0.374	0.87	0.37, 2.04	0.741
1-2	1.28	0.70, 2.32	0.423	1.31	0.70, 2.45	0.390	1.26	0.63, 2.51	0.512
2-3	1.05	0.48, 2.29	0.902	1.18	0.54, 2.61	0.677	1.21	0.52, 2.81	0.662
3-4	0.78	0.24, 2.58	0.685	0.95	0.28, 3.16	0.928	1.27	0.37, 4.31	0.705
4-5	3.07	1.26, 7.46	0.013	2.95	1.02, 8.49	0.045	3.84	1.31, 11.25	0.014
5-6	0.00	NE	0.982	0.00	NE	0.987	0.00	NE	0.988
>6	0.00	NE	0.986	0.00	NE	0.994	0.00	NE	0.996
**Bladder cancer**									
**Glargine vs Human insulin**									
0-0.5	1.41	0.92, 2.17	0.118	1.32	0.82, 2.13	0.249	1.32	0.82, 2.13	0.260
0.5-1	0.97	0.57, 1.65	0.902	0.95	0.54, 1.70	0.875	0.89	0.49, 1.64	0.714
1-2	0.76	0.46, 1.25	0.273	0.61	0.34, 1.10	0.101	0.70	0.39, 1.28	0.248
2-3	0.93	0.56, 1.52	0.764	0.97	0.57, 1.65	0.904	1.02	0.58, 1.80	0.951
3-4	0.87	0.48, 1.56	0.630	0.93	0.48, 1.79	0.819	0.86	0.42, 1.76	0.672
4-5	1.68	0.87, 3.25	0.125	1.48	0.69, 3.17	0.316	1.16	0.49, 2.73	0.734

Cumulative treatment time, years	Type 2 diabetes			Nordic population			Entry from 2000 onward		
	RR	95% CI	P	RR	95% CI	P	RR	95% CI	P
5-6	0.67	0.20, 2.25	0.514	0.74	0.17, 3.24	0.693	0.34	0.05, 2.59	0.300
>6	0.59	0.18, 1.92	0.380	0.73	0.17, 3.06	0.662	1.13	0.26, 4.88	0.872
**Glargine vs Detemir**									
0-0.5	1.03	0.55, 1.95	0.923	1.14	0.54, 2.40	0.722	1.27	0.59, 2.75	0.540
0.5-1	1.08	0.49, 2.38	0.858	0.94	0.41, 2.12	0.877	0.82	0.36, 1.90	0.650
1-2	0.55	0.29, 1.04	0.067	0.46	0.22, 0.96	0.038	0.57	0.26, 1.23	0.154
2-3	0.96	0.44, 2.08	0.917	0.94	0.41, 2.16	0.892	0.96	0.40, 2.32	0.931
3-4	0.63	0.26, 1.55	0.313	0.67	0.24, 1.92	0.459	0.69	0.22, 2.22	0.536
4-5	1.56	0.35, 6.93	0.559	0.86	0.19, 4.00	0.850	0.66	0.14, 3.18	0.603
5-6	NE	NE	0.981	NE	NE	0.987	NE	NE	0.989
>6	NE	NE	0.986	NE	NE	0.995	NE	NE	0.996
**Detemir vs Human Insulin**									
0-0.5	1.37	0.76, 2.46	0.300	1.16	0.58, 2.29	0.677	1.04	0.51, 2.12	0.924
0.5-1	0.90	0.43, 1.86	0.775	1.02	0.49, 2.13	0.961	1.08	0.51, 2.28	0.834
1-2	1.36	0.79, 2.36	0.264	1.32	0.73, 2.40	0.359	1.23	0.64, 2.38	0.536
2-3	0.97	0.47, 1.98	0.924	1.02	0.48, 2.21	0.950	1.06	0.47, 2.41	0.892
3-4	1.38	0.61, 3.08	0.439	1.38	0.53, 3.54	0.509	1.24	0.43, 3.54	0.694
4-5	1.08	0.25, 4.54	0.921	1.71	0.40, 7.27	0.465	1.76	0.41, 7.56	0.445
5-6	0.00	NE	0.981	0.00	NE	0.986	0.00	NE	0.988
>6	0.00	NE	0.986	0.00	NE	0.995	0.00	NE	0.996
**Colorectal cancer**									
**Glargine vs Human insulin**									
0-0.5	1.26	0.92, 1.73	0.145	1.16	0.82, 1.64	0.408	1.09	0.76, 1.57	0.633
0.5-1	0.85	0.57, 1.26	0.411	0.89	0.58, 1.38	0.613	0.90	0.57, 1.42	0.646
1-2	0.81	0.58, 1.13	0.208	0.67	0.45, 0.98	0.039	0.63	0.42, 0.96	0.032
2-3	1.09	0.77, 1.54	0.617	1.02	0.69, 1.50	0.930	1.06	0.71, 1.59	0.778
3-4	0.86	0.54, 1.37	0.519	0.74	0.43, 1.29	0.289	0.81	0.46, 1.44	0.482
4-5	1.04	0.59, 1.81	0.903	0.80	0.39, 1.62	0.531	0.80	0.35, 1.79	0.582
5-6	0.68	0.29, 1.59	0.374	0.38	0.09, 1.58	0.183	0.26	0.03, 1.88	0.180
>6	0.51	0.23, 1.10	0.085	0.62	0.23, 1.72	0.362	0.38	0.09, 1.57	0.183
**Glargine vs Detemir**									
0-0.5	1.41	0.84, 2.37	0.189	1.31	0.74, 2.31	0.350	1.54	0.82, 2.90	0.181
0.5-1	1.10	0.61, 1.99	0.743	1.10	0.58, 2.07	0.770	0.96	0.50, 1.83	0.902
1-2	1.11	0.68, 1.80	0.674	0.96	0.55, 1.68	0.894	0.78	0.44, 1.39	0.403
2-3	1.17	0.68, 2.02	0.571	1.01	0.56, 1.82	0.972	1.12	0.59, 2.12	0.737
3-4	0.84	0.39, 1.82	0.655	0.72	0.29, 1.75	0.464	0.64	0.26, 1.58	0.338
4-5	0.91	0.33, 2.50	0.860	0.53	0.16, 1.74	0.297	0.52	0.14, 2.03	0.351
5-6	0.72	0.14, 3.55	0.682	NE	NE	0.980	NE	NE	0.982
>6	NE	NE	0.966	NE	NE	0.991	NE	NE	0.993
**Detemir vs Human Insulin**									
0-0.5	0.89	0.55, 1.44	0.639	0.88	0.53, 1.48	0.639	0.71	0.40, 1.27	0.248
0.5-1	0.77	0.45, 1.30	0.322	0.81	0.46, 1.43	0.473	0.94	0.53, 1.65	0.819

Cumulative treatment time, years	Type 2 diabetes			Nordic population			Entry from 2000 onward		
	RR	95% CI	P	RR	95% CI	P	RR	95% CI	P
1-2	0.73	0.47, 1.14	0.162	0.69	0.42, 1.13	0.141	0.81	0.49, 1.33	0.404
2-3	0.93	0.56, 1.55	0.788	1.01	0.59, 1.73	0.981	0.95	0.52, 1.72	0.865
3-4	1.02	0.51, 2.05	0.949	1.04	0.47, 2.27	0.925	1.27	0.57, 2.79	0.560
4-5	1.13	0.45, 2.84	0.790	1.49	0.54, 4.15	0.443	1.52	0.47, 4.94	0.488
5-6	0.95	0.23, 3.94	0.943	0.00	NE	0.978	0.00	NE	0.980
>6	0.00	, NE	0.965	0.00	NE	0.991	0.00	NE	0.993
**Non-Hodgkin's lymphoma**									
**Glargine vs Human insulin**									
0-0.5	0.66	0.34, 1.30	0.230	0.56	0.27, 1.17	0.122	0.55	0.26, 1.14	0.108
0.5-1	0.96	0.46, 2.00	0.908	0.97	0.45, 2.09	0.929	0.95	0.38, 2.37	0.919
1-2	1.03	0.49, 2.17	0.939	1.13	0.51, 2.48	0.768	1.12	0.51, 2.49	0.772
2-3	0.51	0.19, 1.36	0.175	0.61	0.23, 1.68	0.342	0.77	0.27, 2.17	0.621
3-4	1.21	0.58, 2.55	0.610	0.93	0.41, 2.14	0.868	0.82	0.34, 1.96	0.649
4-5	1.39	0.57, 3.36	0.471	0.89	0.30, 2.67	0.834	0.93	0.31, 2.81	0.894
5-6	0.90	0.20, 4.00	0.889	0.00	NE	0.982	0.00	NE	0.984
>6	1.56	0.58, 4.19	0.378	1.84	0.62, 5.46	0.274	1.67	0.48, 5.83	0.425
**Glargine vs Detemir**									
0-0.5	1.28	0.41, 4.07	0.671	1.04	0.32, 3.42	0.944	1.06	0.32, 3.47	0.927
0.5-1	0.80	0.31, 2.09	0.648	0.73	0.27, 1.93	0.522	0.51	0.18, 1.48	0.218
1-2	0.47	0.20, 1.11	0.084	0.47	0.19, 1.15	0.099	0.53	0.21, 1.33	0.175
2-3	0.24	0.08, 0.73	0.012	0.23	0.08, 0.72	0.011	0.24	0.08, 0.73	0.012
3-4	NE	NE	0.964	NE	NE	0.980	NE	NE	0.981
4-5	NE	NE	0.973	NE	NE	0.986	NE	NE	0.987
5-6	NE	NE	0.982	0.29	NE	0.999	0.29	NE	0.999
>6	NE	NE	0.986	NE	NE	0.996	NE	NE	0.997
**Detemir vs Human Insulin**									
0-0.5	0.51	0.18, 1.46	0.212	0.54	0.19, 1.53	0.244	0.52	0.18, 1.47	0.217
0.5-1	1.20	0.50, 2.87	0.686	1.33	0.55, 3.22	0.527	1.86	0.74, 4.68	0.189
1-2	2.18	1.02, 4.64	0.044	2.39	1.07, 5.33	0.034	2.12	0.92, 4.88	0.078
2-3	2.13	0.92, 4.94	0.078	2.62	1.11, 6.22	0.028	3.25	1.32, 7.97	0.010
3-4	0.00	NE	0.964	0.00	NE	0.980	0.00	NE	0.981
4-5	0.00	NE	0.974	0.00	NE	0.986	0.00	NE	0.987
5-6	0.00	NE	0.982	0.00	NE	0.991	0.00	NE	0.992
>6	0.00	NE	0.987	0.00	NE	0.996	0.00	NE	0.997
**Prostate cancer**									
**Glargine vs Human insulin**									
0-0.5	1.07	0.86, 1.34	0.528	1.04	0.82, 1.32	0.737	1.03	0.81, 1.32	0.793
0.5-1	0.93	0.71, 1.22	0.595	0.91	0.68, 1.22	0.525	0.96	0.71, 1.30	0.807
1-2	1.00	0.79, 1.27	0.979	1.02	0.80, 1.31	0.874	1.04	0.8, 1.35	0.758
2-3	0.91	0.69, 1.18	0.463	0.82	0.61, 1.10	0.178	0.74	0.54, 1.03	0.079
3-4	0.98	0.72, 1.34	0.893	0.95	0.68, 1.34	0.777	0.79	0.54, 1.15	0.214
4-5	0.86	0.57, 1.29	0.462	0.93	0.60, 1.43	0.730	0.82	0.51, 1.34	0.430

Cumulative treatment time, years	Type 2 diabetes			Nordic population			Entry from 2000 onward		
	RR	95% CI	P	RR	95% CI	P	RR	95% CI	P
5-6	0.91	0.55, 1.50	0.711	0.75	0.40, 1.41	0.372	0.57	0.26, 1.24	0.156
>6	0.82	0.51, 1.32	0.411	0.94	0.54, 1.63	0.833	0.91	0.46, 1.80	0.784
**Glargine vs Detemir**									
0-0.5	1.11	0.79, 1.56	0.534	1.11	0.77, 1.60	0.586	1.13	0.77, 1.65	0.532
0.5-1	1.10	0.75, 1.63	0.617	1.02	0.68, 1.54	0.921	1.02	0.67, 1.57	0.920
1-2	1.04	0.75, 1.45	0.816	1.06	0.74, 1.51	0.747	0.99	0.68, 1.43	0.953
2-3	1.03	0.68, 1.58	0.881	0.90	0.57, 1.42	0.664	0.77	0.47, 1.25	0.292
3-4	0.75	0.46, 1.23	0.259	0.70	0.41, 1.20	0.193	0.71	0.38, 1.33	0.287
4-5	1.56	0.55, 4.46	0.402	1.18	0.41, 3.38	0.762	0.86	0.30, 2.53	0.791
5-6	0.69	0.23, 2.06	0.511	0.35	0.1, 1.270	0.110	0.20	0.05, 0.76	0.018
>6	0.94	0.22, 4.06	0.937	NE	NE	0.953	NE	NE	0.964
**Detemir vs Human Insulin**									
0-0.5	0.96	0.71, 1.32	0.821	0.94	0.67, 1.31	0.720	0.92	0.65, 1.29	0.618
0.5-1	0.84	0.59, 1.20	0.346	0.89	0.61, 1.30	0.548	0.94	0.64, 1.39	0.766
1-2	0.96	0.71, 1.32	0.820	0.96	0.69, 1.34	0.821	1.05	0.75, 1.49	0.766
2-3	0.88	0.59, 1.30	0.510	0.90	0.6, 1.370	0.629	0.97	0.62, 1.50	0.890
3-4	1.30	0.83, 2.05	0.255	1.36	0.83, 2.21	0.217	1.10	0.63, 1.93	0.731
4-5	0.55	0.20, 1.49	0.239	0.79	0.29, 2.14	0.640	0.95	0.35, 2.60	0.921
5-6	1.31	0.48, 3.57	0.598	2.13	0.67, 6.77	0.199	2.92	0.92, 9.31	0.070
>6	0.87	0.21, 3.52	0.843	0.00	NE	0.953	0.00	NE	0.963
**Any cancer [a]**									
**Glargine vs Human insulin**									
0-0.5	1.09	0.99, 1.20	0.095	1.06	0.95, 1.18	0.310	1.04	0.93, 1.16	0.513
0.5-1	0.88	0.77, 1.00	0.052	0.90	0.78, 1.04	0.141	0.91	0.78, 1.05	0.205
1-2	0.89	0.80, 1.00	0.053	0.84	0.74, 0.95	0.006	0.84	0.73, 0.96	0.011
2-3	0.84	0.73, 0.95	0.008	0.82	0.71, 0.94	0.006	0.81	0.69, 0.95	0.008
3-4	0.91	0.78, 1.06	0.234	0.90	0.75, 1.06	0.211	0.86	0.71, 1.04	0.112
4-5	0.94	0.78, 1.14	0.528	0.83	0.66, 1.04	0.104	0.73	0.56, 0.94	0.017
5-6	0.94	0.73, 1.20	0.617	0.80	0.58, 1.10	0.176	0.82	0.58, 1.16	0.263
>6	0.63	0.50, 0.80	0.000	0.74	0.55, 0.99	0.042	0.77	0.54, 1.08	0.127
**Glargine vs Detemir**									
0-0.5	1.23	1.05, 1.43	0.009	1.24	1.04, 1.46	0.014	1.26	1.06, 1.50	0.010
0.5-1	1.05	0.87, 1.27	0.591	1.09	0.89, 1.34	0.407	1.02	0.82, 1.26	0.877
1-2	1.01	0.86, 1.18	0.934	0.99	0.83, 1.18	0.892	0.92	0.76, 1.11	0.382
2-3	1.05	0.85, 1.30	0.628	0.97	0.77, 1.21	0.757	0.94	0.74, 1.20	0.611
3-4	0.86	0.67, 1.11	0.240	0.78	0.59, 1.04	0.089	0.85	0.62, 1.17	0.318
4-5	1.03	0.70, 1.51	0.881	0.79	0.50, 1.25	0.309	0.63	0.39, 1.03	0.068
5-6	1.00	0.57, 1.74	0.994	1.02	0.40, 2.57	0.969	0.75	0.29, 1.91	0.546
>6	5.14	1.26, 20.93	0.022	NE	NE	0.923	NE	NE	0.939
**Detemir vs Human Insulin**									
0-0.5	0.89	0.77, 1.02	0.093	0.86	0.73, 1.00	0.050	0.82	0.70, 0.97	0.019
0.5-1	0.84	0.71, 0.99	0.037	0.83	0.69, 0.99	0.041	0.89	0.74, 1.08	0.243

Cumulative treatment time, years	Type 2 diabetes			Nordic population			Entry from 2000 onward		
	RR	95% CI	P	RR	95% CI	P	RR	95% CI	P
1-2	0.89	0.77, 1.03	0.115	0.85	0.72, 1.00	0.048	0.91	0.77, 1.08	0.291
2-3	0.80	0.66, 0.96	0.020	0.85	0.69, 1.04	0.108	0.86	0.69, 1.08	0.189
3-4	1.06	0.84, 1.34	0.623	1.14	0.89, 1.48	0.302	1.01	0.76, 1.35	0.944
4-5	0.91	0.64, 1.30	0.618	1.05	0.69, 1.60	0.820	1.15	0.74, 1.79	0.536
5-6	0.94	0.56, 1.58	0.818	0.79	0.33, 1.91	0.598	1.09	0.45, 2.65	0.843
>6	0.12	0.03, 0.49	0.003	0.00	NE	0.921	0.00	NE	0.938

[a] Any cancer except non-melanoma skin cancer

Abbreviations: RR – rate ratio, CI – confidence interval, NE – not estimable.

ESM Table 3. Results of the sensitivity analyses for women: adjusted (all sites and any cancer: age, calendar time, NIADs, duration of insulin-treated diabetes, country; liver, colorectal, breast, and endometrial cancer: additional adjustment for relevant co-medications)rate ratios (RR) with 95% confidence interval (CI) from four sensitivity analyses for site-specific cancers and any cancer. Pairwise comparisons of glargine, detemir, and human insulin by the cumulative treatment time (years), when restricting the study population (type 2 diabetes, or Nordic countries, or study entry in 2000 or later), or using additional adjustment (menopause status for breast and endometrial cancer).

Cumulative treatment time	Type 2 diabetes			Nordic population			Entry from 2000 onward			Adjusted for menopause		
	RR	95% CI	P	RR	95% CI	P	RR	95% CI	P	RR	95% CI	P
**Liver camcer**												
**Glargine vs Human insulin**												
0-0.5	1.04	0.46, 2.34	0.931	0.93	0.36, 2.38	0.881	0.78	0.29, 2.11	0.626			
0.5-1	0.46	0.14, 1.48	0.192	0.40	0.11, 1.52	0.179	0.36	0.07, 1.74	0.203			
1-2	0.62	0.20, 1.89	0.399	0.41	0.11, 1.61	0.202	0.43	0.11, 1.65	0.217			
2-3	0.60	0.20, 1.82	0.371	0.71	0.22, 2.24	0.557	0.85	0.26, 2.76	0.785			
3-4	0.51	0.16, 1.64	0.259	0.57	0.17, 1.86	0.349	0.82	0.24, 2.82	0.755			
4-5	0.73	0.20, 2.75	0.646	0.85	0.22, 3.32	0.816	0.59	0.12, 2.81	0.503			
5-6	0.00	0.00, 0.00	NE	0.00	NE	0.984	0.00	NE	0.986			
>6	0.57	0.12, 2.63	0.472	0.79	0.16, 3.83	0.772	1.65	0.33, 8.20	0.540			
**Glargine vs Detemir**												
0-0.5	1.65	0.45, 6.12	0.453	1.75	0.36, 8.56	0.492	1.48	0.29, 7.50	0.633			
0.5-1	1.11	0.20, 6.16	0.907	0.86	0.14, 5.25	0.871	1.15	0.1, 12.85	0.912			
1-2	0.64	0.17, 2.45	0.514	0.53	0.1, 2.7	0.447	0.79	0.13, 4.87	0.802			
2-3	1.06	0.20, 5.57	0.943	1.09	0.21, 5.71	0.922	1.09	0.21, 5.72	0.922			
3-4	0.67	0.12, 3.72	0.649	NE	NE	0.981	NE	NE	0.982			
4-5	NE	NE	0.984	NE	NE	0.986	NE	NE	0.988			
5-6	0.58	NE	1.000	0.49	NE	1.000	0.43	NE	1.000			
>6	NE	NE	0.992	NE	NE	0.996	NE	NE	0.997			
**Detemir vs Human Insulin**												
0-0.5	0.63	0.18, 2.22	0.470	0.53	0.12, 2.43	0.416	0.53	0.12, 2.41	0.408			

Cumulative treatment time	Type 2 diabetes			Nordic population			Entry from 2000 onward			Adjusted for menopause		
	RR	95% CI	P	RR	95% CI	P	RR	95% CI	P	RR	95% CI	P
0.5-1	0.41	0.09, 1.93	0.261	0.47	0.1, 2.24	0.341	0.31	0.04, 2.59	0.281			
1-2	0.97	0.29, 3.26	0.956	0.78	0.2, 3.08	0.718	0.54	0.11, 2.64	0.445			
2-3	0.57	0.12, 2.70	0.477	0.65	0.13, 3.22	0.599	0.78	0.15, 3.95	0.765			
3-4	0.76	0.16, 3.55	0.729	0.00	NE	0.980	0.00	NE	0.982			
4-5	0.00	NE	0.983	0.00	NE	0.986	0.00	NE	0.987			
5-6	0.00	NE	0.988	0.00	NE	0.991	0.00	NE	0.993			
>6	0.00	NE	0.992	0.00	NE	0.996	0.00	NE	0.997			
**Pancreatic cancer**												
**Glargine vs Human insulin**												
0-0.5	0.85	0.64, 1.12	0.243	0.84	0.61, 1.14	0.263	0.80	0.59, 1.10	0.172			
0.5-1	0.99	0.65, 1.50	0.947	1.12	0.72, 1.73	0.622	1.23	0.78, 1.94	0.380			
1-2	1.00	0.65, 1.53	0.995	0.88	0.54, 1.42	0.598	1.03	0.62, 1.71	0.909			
2-3	1.36	0.74, 2.47	0.320	1.42	0.75, 2.67	0.282	1.07	0.50, 2.30	0.866			
3-4	0.75	0.31, 1.84	0.532	0.85	0.35, 2.10	0.729	1.56	0.59, 4.11	0.366			
4-5	1.29	0.51, 3.23	0.592	1.58	0.62, 4.01	0.338	1.67	0.59, 4.71	0.334			
5-6	0.62	0.14, 2.69	0.526	0.43	0.06, 3.23	0.409	0.56	0.07, 4.34	0.579			
>6	1.02	0.31, 3.35	0.973	0.97	0.23, 4.12	0.972	0.61	0.08, 4.54	0.627			
**Glargine vs Detemir**												
0-0.5	1.17	0.75, 1.82	0.484	1.39	0.83, 2.33	0.214	1.33	0.79, 2.24	0.283			
0.5-1	1.28	0.65, 2.49	0.477	1.20	0.61, 2.37	0.599	1.14	0.58, 2.27	0.700			
1-2	0.65	0.37, 1.13	0.129	0.51	0.28, 0.94	0.030	0.48	0.26, 0.89	0.019			
2-3	1.55	0.51, 4.66	0.436	1.39	0.45, 4.24	0.568	1.15	0.31, 4.25	0.840			
3-4	NE	NE	0.966	NE	NE	0.970	NE	NE	0.972			
4-5	0.82	0.16, 4.09	0.809	0.72	0.14, 3.62	0.694	1.17	0.14, 10.08	0.889			
5-6	NE	NE	0.982	NE	NE	0.987	NE	NE	0.988			
>6	0.40	0.04, 3.89	0.431	NE	NE	0.995	NE	NE	0.996			
**Detemir vs Human Insulin**												

Cumulative treatment time	Type 2 diabetes			Nordic population			Entry from 2000 onward			Adjusted for menopause		
	RR	95% CI	P	RR	95% CI	P	RR	95% CI	P	RR	95% CI	P
0-0.5	0.72	0.48, 1.08	0.115	0.60	0.37, 0.97	0.038	0.60	0.37, 0.97	0.039			
0.5-1	0.77	0.42, 1.43	0.412	0.93	0.50, 1.73	0.821	1.07	0.57, 2.02	0.826			
1-2	1.54	0.94, 2.51	0.084	1.71	1.03, 2.84	0.037	2.14	1.27, 3.61	0.004			
2-3	0.88	0.31, 2.49	0.803	1.02	0.36, 2.92	0.967	0.93	0.28, 3.11	0.910			
3-4	0.00	NE	0.965	0.00	NE	0.970	0.00	NE	0.973			
4-5	1.57	0.36, 6.79	0.547	2.18	0.5, 9.56	0.301	1.43	0.18, 11.10	0.732			
5-6	0.00	NE	0.981	0.00	NE	0.986	0.00	NE	0.988			
>6	2.54	0.34, 18.83	0.363	0.00	NE	0.995	0.00	NE	0.995			
**Lung cancer**												
**Glargine vs Human insulin**												
0-0.5	1.27	0.83, 1.95	0.278	1.34	0.84, 2.15	0.221	1.18	0.72, 1.93	0.502			
0.5-1	1.21	0.67, 2.18	0.530	1.45	0.76, 2.78	0.265	1.44	0.71, 2.91	0.310			
1-2	0.84	0.52, 1.37	0.488	0.69	0.37, 1.29	0.247	0.72	0.36, 1.42	0.340			
2-3	0.75	0.40, 1.40	0.364	0.55	0.24, 1.24	0.152	0.41	0.16, 1.06	0.065			
3-4	1.08	0.52, 2.24	0.828	1.46	0.65, 3.29	0.360	1.42	0.59, 3.38	0.433			
4-5	2.09	1.01, 4.34	0.048	2.83	1.31, 6.09	0.008	2.86	1.27, 6.44	0.011			
5-6	1.68	0.72, 3.92	0.231	1.33	0.39, 4.49	0.646	0.68	0.09, 5.18	0.712			
>6	0.47	0.14, 1.53	0.208	0.97	0.23, 4.10	0.966	1.20	0.28, 5.11	0.809			
**Glargine vs Detemir**												
0-0.5	0.98	0.54, 1.77	0.935	0.99	0.51, 1.92	0.978	0.92	0.46, 1.82	0.812			
0.5-1	1.34	0.57, 3.15	0.501	1.34	0.53, 3.37	0.536	1.25	0.46, 3.39	0.657			
1-2	1.99	0.85, 4.69	0.114	1.29	0.48, 3.45	0.607	1.22	0.42, 3.55	0.719			
2-3	0.69	0.29, 1.63	0.399	0.69	0.22, 2.20	0.532	0.56	0.15, 2.11	0.392			
3-4	0.52	0.20, 1.33	0.174	0.52	0.18, 1.52	0.232	0.41	0.14, 1.22	0.109			
4-5	NE	NE	0.958	NE	NE	0.965	NE	NE	0.967			
5-6	2.27	0.28, 18.52	0.445	NE	NE	0.978	NE	NE	0.982			
>6	0.60	0.06, 5.82	0.662	NE	NE	0.991	NE	NE	0.993			

Cumulative treatment time	Type 2 diabetes			Nordic population			Entry from 2000 onward			Adjusted for menopause		
	RR	95% CI	P	RR	95% CI	P	RR	95% CI	P	RR	95% CI	P
**Detemir vs Human Insulin**												
0-0.5	1.30	0.77, 2.21	0.332	1.36	0.76, 2.42	0.304	1.28	0.71, 2.33	0.411			
0.5-1	0.90	0.41, 1.97	0.794	1.08	0.47, 2.51	0.855	1.15	0.46, 2.85	0.765			
1-2	0.42	0.19, 0.94	0.034	0.53	0.22, 1.27	0.155	0.59	0.23, 1.52	0.274			
2-3	1.08	0.52, 2.26	0.829	0.80	0.31, 2.06	0.641	0.73	0.26, 2.09	0.561			
3-4	2.08	0.94, 4.62	0.073	2.80	1.12, 7.01	0.027	3.48	1.37, 8.83	0.009			
4-5	0.00	NE	0.960	0.00	NE	0.967	0.00	NE	0.970			
5-6	0.74	0.10, 5.52	0.769	0.00	NE	0.979	0.00	NE	0.981			
>6	0.78	0.11, 5.72	0.804	0.00	NE	0.991	0.00	NE	0.993			
**Melanoma of skin**												
**Glargine vs Human insulin**												
0-0.5	1.58	0.88, 2.82	0.126	1.62	0.89, 2.92	0.113	1.52	0.82, 2.83	0.182			
0.5-1	1.15	0.59, 2.24	0.688	1.07	0.54, 2.10	0.849	1.22	0.61, 2.44	0.567			
1-2	1.28	0.70, 2.32	0.420	1.21	0.65, 2.24	0.552	1.30	0.68, 2.46	0.423			
2-3	1.81	0.95, 3.44	0.071	1.58	0.81, 3.12	0.182	1.26	0.61, 2.57	0.532			
3-4	0.72	0.29, 1.79	0.477	0.50	0.17, 1.47	0.205	0.46	0.13, 1.60	0.224			
4-5	3.60	1.70, 7.59	0.001	3.74	1.74, 8.02	0.001	4.63	1.88, 11.43	0.001			
5-6	1.18	0.34, 4.08	0.792	0.85	0.19, 3.71	0.824	1.08	0.24, 4.86	0.916			
>6	1.50	0.51, 4.37	0.461	1.39	0.41, 4.74	0.594	2.86	0.8, 10.26	0.107			
**Glargine vs Detemir**												
0-0.5	0.93	0.44, 1.98	0.855	1.05	0.48, 2.30	0.900	0.93	0.42, 2.07	0.860			
0.5-1	1.72	0.61, 4.80	0.303	2.24	0.73, 6.85	0.159	4.40	0.99, 19.53	0.051			
1-2	1.23	0.53, 2.85	0.623	1.37	0.56, 3.32	0.488	1.74	0.63, 4.78	0.281			
2-3	1.80	0.60, 5.39	0.291	1.64	0.54, 4.97	0.380	1.68	0.47, 5.94	0.424			
3-4	1.87	0.22, 15.65	0.562	1.25	0.14, 11.25	0.843	0.88	0.09, 8.55	0.915			
4-5	0.96	0.27, 3.41	0.950	1.31	0.29, 5.89	0.722	0.94	0.20, 4.34	0.935			
5-6	0.54	0.06, 5.25	0.595	0.28	0.02, 3.16	0.304	0.22	0.02, 2.51	0.223			

Cumulative treatment time	Type 2 diabetes			Nordic population			Entry from 2000 onward			Adjusted for menopause		
	RR	95% CI	P	RR	95% CI	P	RR	95% CI	P	RR	95% CI	P
>6	NE	NE	0.971	NE	NE	0.971	NE	NE	0.976			
**Detemir vs Human Insulin**												
0-0.5	1.69	0.83, 3.45	0.149	1.54	0.73, 3.24	0.260	1.64	0.77, 3.48	0.199			
0.5-1	0.67	0.25, 1.80	0.424	0.48	0.16, 1.42	0.183	0.28	0.06, 1.21	0.088			
1-2	1.04	0.46, 2.33	0.934	0.88	0.37, 2.09	0.776	0.75	0.28, 2.01	0.561			
2-3	1.00	0.34, 2.98	0.995	0.97	0.32, 2.89	0.950	0.75	0.22, 2.58	0.648			
3-4	0.38	0.05, 2.88	0.351	0.40	0.05, 3.00	0.371	0.52	0.07, 4.01	0.534			
4-5	3.75	1.06, 13.18	0.040	2.85	0.63, 12.8	0.172	4.94	1.05, 23.29	0.044			
5-6	2.19	0.28, 16.88	0.452	3.01	0.39, 23.46	0.293	4.91	0.62, 39.00	0.132			
>6	0.00	NE	0.972	0.00	NE	0.972	0.00	NE	0.978			
**Bladder cancer**												
**Glargine vs Human insulin**												
0-0.5	1.51	0.64, 3.56	0.345	1.20	0.48, 2.96	0.701	1.53	0.60, 3.92	0.374			
0.5-1	2.96	0.91, 9.64	0.072	2.57	0.77, 8.54	0.123	2.52	0.76, 8.32	0.129			
1-2	0.86	0.30, 2.51	0.785	0.74	0.25, 2.21	0.585	0.17	0.02, 1.32	0.089			
2-3	0.66	0.21, 2.04	0.474	0.55	0.15, 2.02	0.370	0.41	0.09, 1.87	0.247			
3-4	0.78	0.16, 3.66	0.749	0.82	0.16, 4.12	0.809	0.80	0.16, 3.97	0.785			
4-5	1.24	0.26, 5.94	0.791	1.00	0.20, 5.07	0.996	1.63	0.31, 8.55	0.562			
5-6	1.97	0.41, 9.47	0.495	0.81	0.10, 6.83	0.843	2.97	0.29, 30.24	0.359			
>6	0.72	0.09, 5.59	0.752	1.19	0.15, 9.74	0.869	0.00	NE	0.992			
**Glargine vs Detemir**												
0-0.5	1.62	0.34, 7.79	0.545	1.53	0.31, 7.56	0.603	3.05	0.37, 25.33	0.303			
0.5-1	1.44	0.28, 7.31	0.658	1.58	0.31, 8.10	0.580	1.53	0.30, 7.83	0.611			
1-2	0.76	0.18, 3.30	0.713	0.81	0.19, 3.56	0.785	0.46	0.03, 7.61	0.591			
2-3	1.41	0.16, 12.82	0.759	1.13	0.12, 11.02	0.919	0.71	0.06, 7.96	0.782			
3-4	0.59	0.05, 6.59	0.668	NE	NE	0.988	NE	NE	0.989			
4-5	NE	NE	0.990	NE	NE	0.991	NE	NE	0.992			

Cumulative treatment time	Type 2 diabetes			Nordic population			Entry from 2000 onward			Adjusted for menopause		
	RR	95% CI	P	RR	95% CI	P	RR	95% CI	P	RR	95% CI	P
5-6	NE	NE	0.992	NE	NE	0.994	NE	NE	0.995			
>6	NE	NE	0.995	NE	NE	0.998	0.18	NE	1.000			
**Detemir vs Human Insulin**												
0-0.5	0.93	0.21, 4.12	0.925	0.78	0.17, 3.50	0.75	0.50	0.06, 3.94	0.51			
0.5-1	2.05	0.39, 10.68	0.394	1.62	0.31, 8.60	0.57	1.65	0.31, 8.73	0.56			
1-2	1.13	0.31, 4.20	0.850	0.90	0.24, 3.44	0.88	0.36	0.04, 2.89	0.33			
2-3	0.47	0.06, 3.65	0.469	0.49	0.06, 3.92	0.50	0.57	0.07, 4.58	0.60			
3-4	1.32	0.16, 10.71	0.797	0.00	NE	0.99	0.00	NE	0.99			
4-5	0.00	NE	0.990	0.00	NE	0.99	0.00	NE	0.99			
5-6	0.00	NE	0.993	0.00	NE	0.99	0.00	NE	1.00			
>6	0.00	NE	0.995	0.00	NE	1.00	0.00	NE	1.00			
**Colorectal cancer**												
**Glargine vs Human insulin**												
0-0.5	1.50	1.03, 2.2	0.036	1.50	1.00, 2.24	0.050	1.31	0.86, 1.99	0.212			
0.5-1	0.80	0.46, 1.40	0.436	0.73	0.40, 1.34	0.309	0.69	0.35, 1.37	0.292			
1-2	1.06	0.69, 1.61	0.793	1.06	0.68, 1.67	0.789	1.08	0.67, 1.74	0.763			
2-3	1.20	0.75, 1.93	0.451	0.91	0.52, 1.58	0.727	1.03	0.56, 1.88	0.923			
3-4	0.63	0.31, 1.29	0.209	0.52	0.22, 1.24	0.141	0.57	0.24, 1.37	0.212			
4-5	1.16	0.58, 2.33	0.682	1.42	0.70, 2.89	0.329	1.87	0.90, 3.89	0.094			
5-6	0.78	0.28, 2.21	0.646	0.81	0.25, 2.66	0.728	0.33	0.04, 2.45	0.278			
>6	0.58	0.21, 1.61	0.295	0.99	0.36, 2.75	0.981	1.00	0.31, 3.27	0.995			
**Glargine vs Detemir**												
0-0.5	1.89	0.96, 3.71	0.064	1.71	0.86, 3.40	0.124	1.48	0.74, 2.96	0.274			
0.5-1	0.44	0.23, 0.84	0.013	0.47	0.23, 0.96	0.038	0.40	0.18, 0.88	0.024			
1-2	1.63	0.79, 3.33	0.183	2.13	0.92, 4.89	0.076	2.05	0.84, 5.05	0.117			
2-3	1.45	0.63, 3.37	0.387	1.13	0.44, 2.89	0.794	1.14	0.41, 3.16	0.800			
3-4	0.35	0.14, 0.93	0.034	0.45	0.13, 1.61	0.219	0.42	0.12, 1.50	0.183			

Cumulative treatment time	Type 2 diabetes			Nordic population			Entry from 2000 onward			Adjusted for menopause		
	RR	95% CI	P	RR	95% CI	P	RR	95% CI	P	RR	95% CI	P
4-5	0.62	0.19, 1.99	0.424	1.09	0.24, 4.98	0.915	1.01	0.22, 4.62	0.992			
5-6	0.29	0.06, 1.31	0.107	0.24	0.04, 1.43	0.116	0.07	0.01, 0.73	0.027			
>6	0.54	0.06, 4.88	0.585	NE	NE	0.994	NE	NE	0.995			
**Detemir vs Human Insulin**												
0-0.5	0.80	0.42, 1.51	0.48	0.87	0.46, 1.67	0.685	0.89	0.46, 1.69	0.716			
0.5-1	1.82	1.08, 3.09	0.03	1.56	0.88, 2.78	0.128	1.74	0.94, 3.22	0.077			
1-2	0.65	0.33, 1.28	0.21	0.50	0.23, 1.11	0.089	0.52	0.22, 1.24	0.140			
2-3	0.83	0.37, 1.84	0.64	0.80	0.34, 1.89	0.609	0.90	0.35, 2.32	0.832			
3-4	1.78	0.83, 3.82	0.14	1.16	0.41, 3.28	0.780	1.36	0.48, 3.88	0.562			
4-5	1.86	0.66, 5.26	0.24	1.31	0.31, 5.50	0.712	1.85	0.44, 7.88	0.403			
5-6	2.70	0.82, 8.90	0.10	3.43	0.81, 14.63	0.095	5.05	1.17, 21.82	0.030			
>6	1.07	0.15, 7.83	0.95	0.00	NE	0.994	0.00	NE	0.995			
**Non, Hodgkin's lymphoma**												
**Glargine vs Human insulin**												
0-0.5	0.69	0.33, 1.44	0.325	0.82	0.39, 1.73	0.606	0.84	0.38, 1.83	0.659			
0.5-1	1.28	0.55, 2.97	0.572	1.24	0.49, 3.14	0.653	1.39	0.54, 3.55	0.497			
1-2	1.00	0.46, 2.14	0.995	0.79	0.33, 1.89	0.598	0.91	0.37, 2.19	0.827			
2-3	1.27	0.59, 2.76	0.543	1.00	0.42, 2.39	0.994	1.04	0.41, 2.65	0.933			
3-4	0.80	0.26, 2.49	0.705	0.83	0.23, 3.00	0.770	1.00	0.27, 3.74	0.997			
4-5	1.05	0.34, 3.26	0.934	0.33	0.04, 2.57	0.291	0.43	0.05, 3.38	0.420			
5-6	1.45	0.29, 7.18	0.648	0.95	0.11, 7.93	0.961	0.92	0.11, 7.76	0.941			
>6	0.29	0.04, 2.22	0.234	0.50	0.06, 3.85	0.506	0.84	0.11, 6.66	0.868			
**Glargine vs Detemir**												
0-0.5	0.80	0.29, 2.24	0.670	0.94	0.31, 2.80	0.907	0.84	0.27, 2.56	0.756			
0.5-1	1.11	0.37, 3.33	0.849	0.87	0.28, 2.74	0.817	0.88	0.28, 2.76	0.825			
1-2	0.75	0.29, 1.99	0.567	0.76	0.24, 2.36	0.630	0.75	0.24, 2.36	0.621			
2-3	1.09	0.34, 3.48	0.878	0.78	0.23, 2.63	0.691	1.35	0.28, 6.58	0.710			

Cumulative treatment time	Type 2 diabetes			Nordic population			Entry from 2000 onward			Adjusted for menopause		
	RR	95% CI	P	RR	95% CI	P	RR	95% CI	P	RR	95% CI	P
3-4	0.64	0.12, 3.52	0.607	0.86	0.09, 8.28	0.894	NE	NE	0.982			
4-5	NE	NE	0.983	NE	NE	0.987	NE	NE	0.988			
5-6	0.46	0.04, 5.13	0.527	0.14	0.01, 2.22	0.161	0.13	0.01, 2.18	0.155			
>6	NE	NE	0.992	NE	NE	0.997	NE	NE	0.997			
**Detemir vs Human Insulin**												
0-0.5	0.87	0.35, 2.13	0.76	0.88	0.33, 2.34	0.793	1.00	0.37, 2.69	1.000			
0.5-1	1.15	0.40, 3.31	0.80	1.42	0.48, 4.20	0.531	1.58	0.53, 4.73	0.416			
1-2	1.32	0.54, 3.25	0.54	1.05	0.37, 2.95	0.931	1.21	0.42, 3.46	0.722			
2-3	1.16	0.38, 3.54	0.79	1.28	0.41, 3.96	0.674	0.77	0.17, 3.49	0.735			
3-4	1.26	0.28, 5.68	0.77	0.96	0.12, 7.60	0.972	0.00	NE	0.982			
4-5	0.00	NE	0.98	0.00	NE	0.986	0.00	NE	0.988			
5-6	3.16	0.37, 26.69	0.29	7.00	0.81, 60.37	0.077	7.20	0.82, 63.09	0.074			
>6	0.00	NE	0.99	0.00	NE	0.997	0.00	NE	0.997			
**Breast cancer**												
**Glargine vs Human insulin**												
0-0.5	1.31	0.97, 1.78	0.076	1.24	0.88, 1.74	0.222	1.31	0.92, 1.87	0.128	1.32	0.98, 1.78	0.070
0.5-1	1.34	0.96, 1.88	0.085	1.21	0.83, 1.76	0.332	1.08	0.72, 1.63	0.717	1.32	0.95, 1.85	0.102
1-2	1.22	0.91, 1.63	0.188	1.14	0.81, 1.58	0.451	1.16	0.81, 1.64	0.416	1.17	0.88, 1.57	0.278
2-3	1.09	0.78, 1.52	0.611	1.15	0.80, 1.66	0.437	1.27	0.86, 1.86	0.232	1.08	0.78, 1.49	0.654
3-4	0.84	0.54, 1.28	0.414	0.85	0.53, 1.37	0.509	0.81	0.49, 1.34	0.412	0.86	0.56, 1.30	0.469
4-5	1.09	0.67, 1.75	0.736	1.00	0.58, 1.72	0.991	1.11	0.61, 2.00	0.731	1.04	0.65, 1.68	0.862
5-6	0.98	0.53, 1.82	0.954	0.63	0.25, 1.58	0.326	0.51	0.16, 1.66	0.266	0.96	0.52, 1.78	0.898
>6	1.13	0.68, 1.89	0.641	1.31	0.68, 2.53	0.425	1.45	0.69, 3.03	0.325	1.10	0.66, 1.84	0.721
**Glargine vs Detemir**												
0-0.5	1.42	0.88, 2.32	0.153	1.15	0.69, 1.93	0.588	1.22	0.71, 2.08	0.469	1.43	0.88, 2.32	0.151
0.5-1	1.33	0.82, 2.15	0.245	1.23	0.72, 2.09	0.447	1.17	0.66, 2.09	0.597	1.28	0.80, 2.05	0.308
1-2	1.00	0.67, 1.49	0.989	1.04	0.66, 1.64	0.867	1.06	0.65, 1.74	0.803	0.95	0.64, 1.41	0.812

Cumulative treatment time	Type 2 diabetes			Nordic population			Entry from 2000 onward			Adjusted for menopause		
	RR	95% CI	P	RR	95% CI	P	RR	95% CI	P	RR	95% CI	P
2-3	1.19	0.70, 2.03	0.517	1.31	0.72, 2.36	0.375	1.30	0.69, 2.45	0.410	1.16	0.69, 1.94	0.582
3-4	0.87	0.43, 1.77	0.703	0.93	0.41, 2.10	0.862	0.89	0.37, 2.13	0.795	0.95	0.47, 1.91	0.883
4-5	0.60	0.29, 1.25	0.175	0.42	0.19, 0.93	0.033	0.39	0.17, 0.90	0.028	0.62	0.30, 1.28	0.196
5-6	0.64	0.22, 1.83	0.404	0.23	0.06, 0.88	0.032	0.16	0.03, 0.81	0.026	0.66	0.23, 1.87	0.432
>6	2.93	0.39, 22.09	0.296	NE	NE	0.952	NE	NE	0.959	2.92	0.39, 22.0	0.298
**Detemir vs Human Insulin**												
0-0.5	0.92	0.59, 1.44	0.722	1.07	0.67, 1.71	0.767	1.08	0.66, 1.76	0.761	0.92	0.59, 1.45	0.733
0.5-1	1.01	0.64, 1.59	0.963	0.98	0.60, 1.61	0.941	0.92	0.54, 1.56	0.766	1.03	0.66, 1.61	0.881
1-2	1.21	0.84, 1.76	0.305	1.09	0.71, 1.67	0.683	1.09	0.69, 1.71	0.721	1.23	0.86, 1.77	0.260
2-3	0.91	0.56, 1.50	0.722	0.88	0.51, 1.54	0.661	0.97	0.53, 1.76	0.920	0.93	0.57, 1.51	0.772
3-4	0.96	0.51, 1.80	0.896	0.92	0.44, 1.91	0.814	0.91	0.41, 1.99	0.807	0.90	0.48, 1.70	0.752
4-5	1.80	0.95, 3.40	0.072	2.37	1.21, 4.65	0.012	2.86	1.40, 5.85	0.004	1.68	0.89, 3.19	0.109
5-6	1.53	0.61, 3.83	0.361	2.69	0.96, 7.53	0.059	3.19	0.97, 10.41	0.055	1.46	0.58, 3.65	0.416
>6	0.39	0.05, 2.77	0.344	0.00	NE	0.953	0.00	NE	0.961	0.38	0.05, 2.70	0.331
**Endometrial cancer**												
**Glargine vs Human insulin**												
0-0.5	1.74	1.05, 2.88	0.031	1.71	1.01, 2.92	0.047	1.60	0.92, 2.78	0.096	1.78	1.07, 2.94	0.026
0.5-1	0.86	0.49, 1.50	0.589	0.86	0.48, 1.56	0.619	0.95	0.52, 1.76	0.79	0.86	0.49, 1.50	0.595
1-2	0.61	0.35, 1.05	0.073	0.61	0.34, 1.08	0.089	0.52	0.27, 0.98	0.044	0.61	0.35, 1.04	0.072
2-3	0.89	0.49, 1.61	0.702	0.69	0.36, 1.34	0.274	0.78	0.39, 1.56	0.476	0.89	0.49, 1.60	0.697
3-4	1.63	0.90, 2.98	0.110	1.46	0.76, 2.78	0.257	1.75	0.88, 3.50	0.112	1.62	0.89, 2.96	0.113
4-5	1.01	0.46, 2.22	0.986	0.87	0.36, 2.11	0.755	0.91	0.34, 2.41	0.846	0.98	0.44, 2.15	0.956
5-6	0.71	0.25, 2.03	0.521	0.66	0.20, 2.21	0.505	0.27	0.04, 1.98	0.196	0.70	0.24, 2.01	0.506
>6	0.36	0.09, 1.47	0.154	0.27	0.04, 2.01	0.203	0.45	0.06, 3.34	0.436	0.34	0.08, 1.42	0.140
**Glargine vs Detemir**												
0-0.5	1.70	0.76, 3.81	0.201	1.48	0.65, 3.38	0.347	1.52	0.63, 3.65	0.348	1.51	0.69, 3.27	0.300
0.5-1	1.97	0.78, 4.99	0.153	1.79	0.69, 4.59	0.229	2.00	0.72, 5.53	0.184	1.97	0.78, 4.99	0.153

Cumulative treatment time	Type 2 diabetes			Nordic population			Entry from 2000 onward			Adjusted for menopause		
	RR	95% CI	P	RR	95% CI	P	RR	95% CI	P	RR	95% CI	P
1-2	1.49	0.62, 3.60	0.372	1.34	0.55, 3.28	0.522	1.01	0.39, 2.56	0.991	1.49	0.62, 3.60	0.371
2-3	0.79	0.35, 1.80	0.573	0.67	0.27, 1.66	0.387	0.68	0.26, 1.78	0.438	0.79	0.35, 1.80	0.575
3-4	1.20	0.44, 3.27	0.726	1.19	0.39, 3.65	0.759	1.43	0.41, 5.07	0.576	1.21	0.44, 3.29	0.716
4-5	1.10	0.23, 5.19	0.907	0.72	0.14, 3.59	0.690	0.59	0.11, 3.04	0.524	1.11	0.24, 5.27	0.891
5-6	NE	NE	0.956	NE	NE	0.968	NE	NE	0.974	NE	NE	0.951
>6	0.13	0.02, 0.91	0.040	NE	NE	0.988	NE	NE	0.990	0.13	0.02, 0.91	0.040
**Detemir vs Human Insulin**												
0-0.5	1.03	0.48, 2.22	0.945	1.16	0.53, 2.51	0.716	1.05	0.46, 2.39	0.906	1.18	0.57, 2.45	0.660
0.5-1	0.44	0.18, 1.04	0.062	0.48	0.20, 1.17	0.105	0.48	0.18, 1.25	0.131	0.44	0.18, 1.04	0.063
1-2	0.41	0.18, 0.92	0.030	0.45	0.20, 1.03	0.058	0.52	0.23, 1.17	0.115	0.41	0.18, 0.91	0.029
2-3	1.13	0.54, 2.37	0.747	1.03	0.47, 2.26	0.941	1.13	0.49, 2.62	0.767	1.13	0.54, 2.36	0.753
3-4	1.36	0.52, 3.56	0.526	1.22	0.42, 3.53	0.712	1.22	0.36, 4.13	0.748	1.35	0.52, 3.52	0.542
4-5	0.92	0.22, 3.89	0.908	1.20	0.28, 5.12	0.802	1.55	0.36, 6.69	0.558	0.88	0.21, 3.72	0.860
5-6	0.00	NE	0.955	0.00	NE	0.967	0.00	NE	0.970	0.00	NE	0.949
>6	2.79	0.66, 11.89	0.164	0.00	NE	0.987	0.00	NE	0.989	2.71	0.64, 11.53	0.177
**Any cancer [a]**												
**Glargine vs Human insulin**												
0-0.5	1.15	1.01, 1.29	0.028	1.12	0.98, 1.28	0.084	1.10	0.96, 1.26	0.185			
0.5-1	1.11	0.95, 1.30	0.180	1.13	0.96, 1.34	0.150	1.20	1.00, 1.43	0.045			
1-2	0.91	0.79, 1.05	0.214	0.87	0.74, 1.01	0.073	0.87	0.73, 1.03	0.106			
2-3	1.03	0.88, 1.21	0.674	0.96	0.80, 1.15	0.675	0.93	0.76, 1.12	0.434			
3-4	0.86	0.70, 1.05	0.136	0.87	0.70, 1.09	0.226	0.91	0.71, 1.15	0.413			
4-5	1.10	0.88, 1.37	0.417	1.11	0.87, 1.43	0.403	1.18	0.90, 1.56	0.229			
5-6	0.96	0.71, 1.29	0.777	0.71	0.47, 1.06	0.092	0.60	0.36, 0.98	0.042			
>6	0.76	0.57, 1.01	0.058	0.96	0.68, 1.36	0.810	1.06	0.71, 1.57	0.787			
**Glargine vs Detemir**												
0-0.5	1.11	0.92, 1.33	0.273	1.07	0.88, 1.31	0.490	1.05	0.86, 1.29	0.614			

Cumulative treatment time	Type 2 diabetes			Nordic population			Entry from 2000 onward			Adjusted for menopause		
	RR	95% CI	P	RR	95% CI	P	RR	95% CI	P	RR	95% CI	P
0.5-1	1.16	0.93, 1.44	0.196	1.17	0.92, 1.48	0.209	1.19	0.92, 1.53	0.187			
1-2	0.96	0.79, 1.17	0.670	0.96	0.77, 1.20	0.696	0.92	0.73, 1.17	0.515			
2-3	1.12	0.86, 1.44	0.398	1.10	0.83, 1.47	0.514	1.06	0.78, 1.46	0.697			
3-4	0.78	0.57, 1.07	0.128	0.96	0.65, 1.42	0.845	0.97	0.64, 1.48	0.903			
4-5	1.10	0.71, 1.71	0.661	0.91	0.55, 1.49	0.702	0.82	0.49, 1.39	0.467			
5-6	0.77	0.44, 1.33	0.343	0.54	0.24, 1.19	0.124	0.35	0.14, 0.84	0.019			
>6	0.84	0.43, 1.66	0.617	NE	NE	0.923	NE	NE	0.935			
**Detemir vs Human Insulin**												
0-0.5	1.03	0.88, 1.22	0.686	1.05	0.88, 1.26	0.605	1.04	0.86, 1.25	0.670			
0.5-1	0.96	0.78, 1.18	0.701	0.97	0.78, 1.21	0.791	1.01	0.80, 1.28	0.921			
1-2	0.95	0.80, 1.14	0.617	0.90	0.74, 1.11	0.332	0.94	0.76, 1.17	0.583			
2-3	0.93	0.73, 1.18	0.531	0.87	0.67, 1.14	0.321	0.87	0.65, 1.16	0.341			
3-4	1.10	0.83, 1.46	0.515	0.91	0.64, 1.29	0.588	0.93	0.63, 1.37	0.711			
4-5	0.99	0.66, 1.50	0.980	1.23	0.78, 1.94	0.381	1.44	0.89, 2.33	0.141			
5-6	1.25	0.76, 2.04	0.375	1.32	0.65, 2.67	0.446	1.72	0.80, 3.67	0.162			
>6	0.9	0.48, 1.69	0.748	0.00	NE	0.923	0.00	NE	0.936			

[a] Any cancer except non-melanoma skin cancer

Abbreviations: RR – rate ratio, CI – confidence interval, NE – not estimable.

ESM Figures

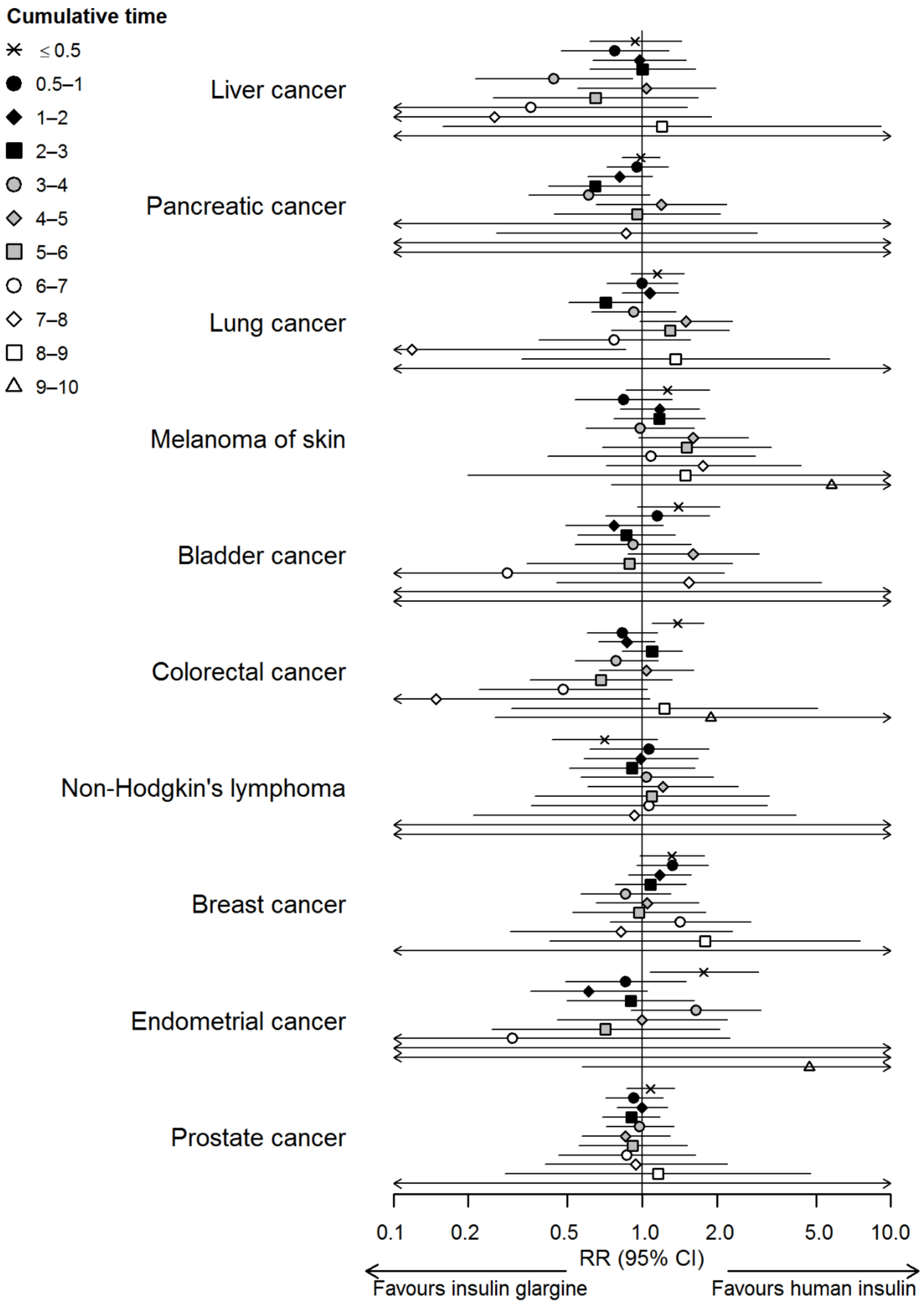

ESM Figure 1. Adjusted (all sites and any cancer: age, calendar time, sex, NIADs, duration of insulin-treated diabetes, country; liver, colorectal, breast, and endometrial cancer: additional adjustment for relevant co-medications) rate ratio (RR) with 95% confidence interval (CI) for site-specific cancers by the cumulative treatment time (years) on glargine versus human insulin.

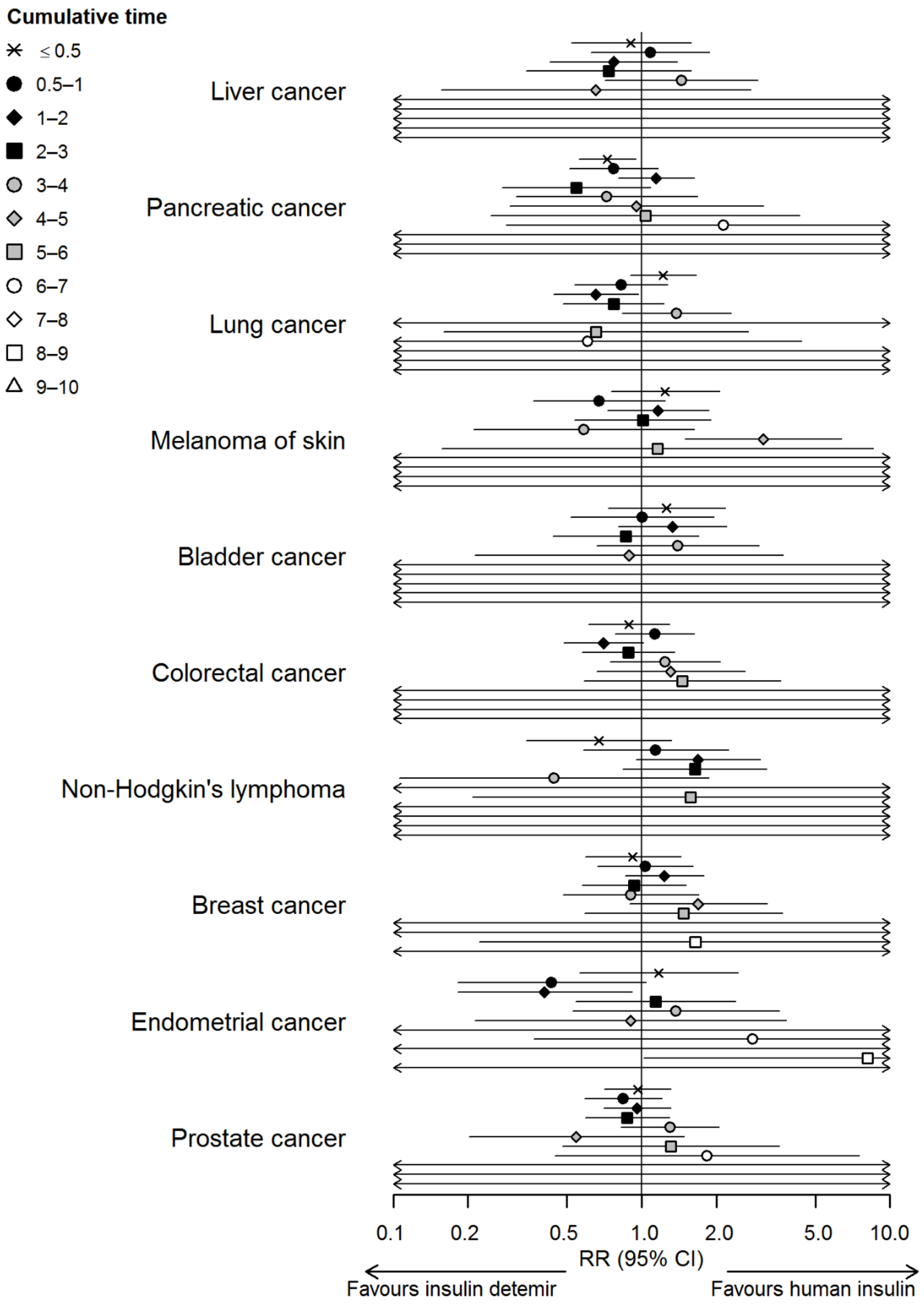

ESM Figure 2. Adjusted (all sites and any cancer: age, calendar time, sex, NIADs, duration of insulin-treated diabetes, country; liver, colorectal, breast, and endometrial cancer: additional adjustment for relevant co-medications) rate ratio (RR) with 95% confidence interval (CI) for site-specific cancers by the cumulative treatment time (years) on detemir versus human insulin.

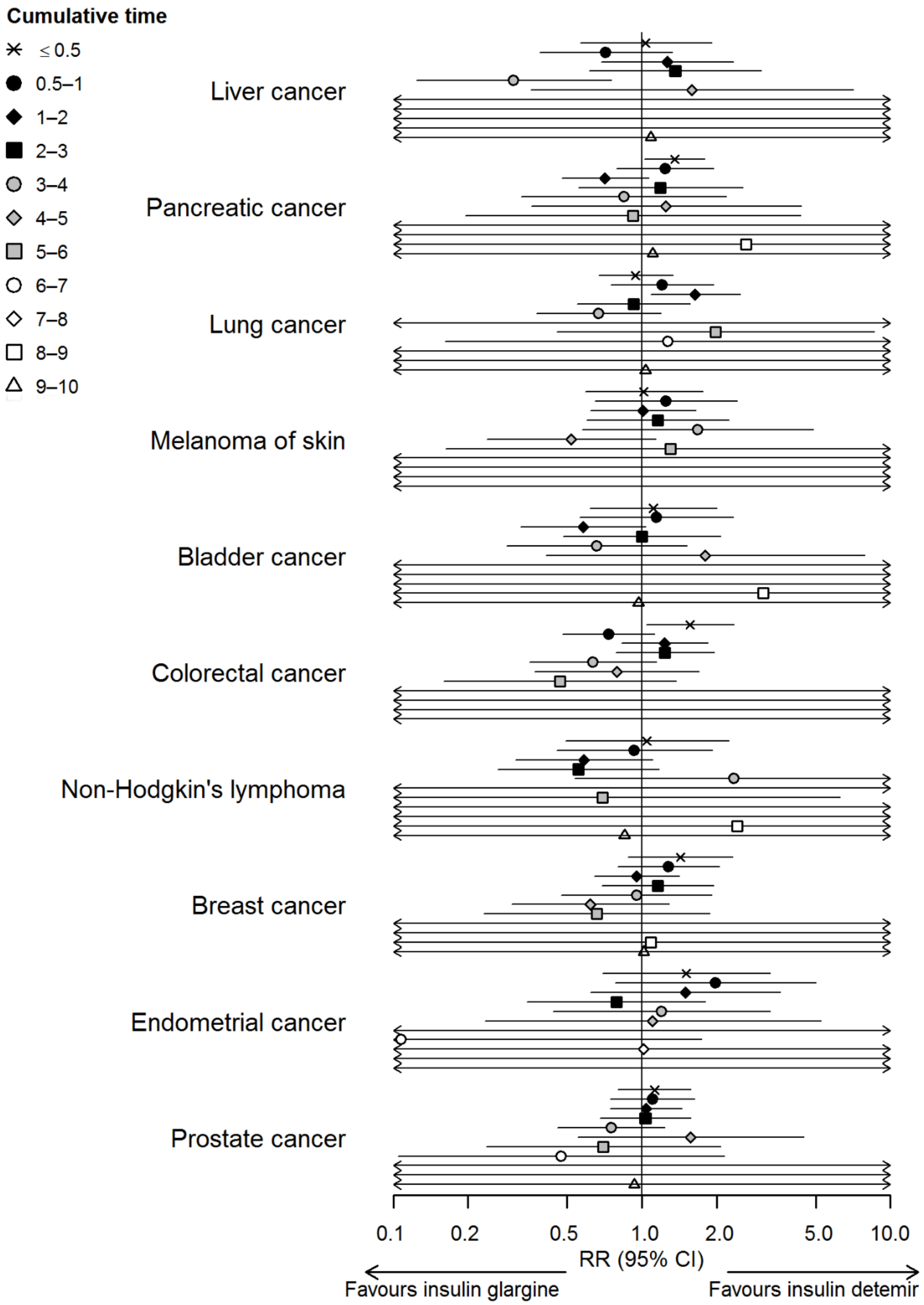

ESM Figure 3. Adjusted (all sites and any cancer: age, calendar time, sex, NIADs, duration of insulin-treated diabetes, country; liver, colorectal, breast, and endometrial cancer: additional adjustment for relevant co-medications) rate ratio (RR) with 95% confidence interval (CI) for site-specific cancers by the cumulative treatment time (years) on glargine versus detemir.
